# Associations of dietary total, heme, non-heme iron intake with diabetes, CVD, and all-cause mortality in men and women with diabetes

**DOI:** 10.1016/j.heliyon.2024.e38758

**Published:** 2024-10-01

**Authors:** Yimin Jin, Yang Huang, Tongshuai Zhang, Qixu Sun, Yao Zhang, Peiru Zhang, Guangyou Wang, Jingyu Zhang, Jinrong Wu

**Affiliations:** a Department of General Practice, The First Affiliated Hospital of Harbin Medical University, Harbin, China; b Wu Lian De Memorial Hospital, The First Affiliated Hospital of Harbin Medical University, Harbin, China; c Department of Neurobiology, Harbin Medical University, Harbin, China; d Ministry of Education Key Laboratory of Preservation of Human Genetic Resources and Disease Control in China, Harbin Medical University, Harbin, China; e Department of Digestive System, YANTAI PENGLAI People's Hospital, Yan Tai, China; f School of Public Health, Harbin Medical University, Harbin, China; g Department of Neurology, The Fourth Affiliated Hospital of Harbin Medical University, Harbin, China; h Department of Anaesthesiology, The First Affiliated Hospital of Harbin Medical University, Harbin, China

**Keywords:** Diabetes, Dietary heme iron, Dietary non-heme iron, Mortality

## Abstract

**Background:**

Iron metabolism disorders significantly increase the risk of diabetes and its related complications by inducing oxidative stress, inflammation, insulin resistance, and disturbances in glucose and lipid metabolism. However, whether dietary iron intake can influence progression of diabetes remains unclear. The present study aims to investigate the relationship between total iron, heme iron, and non-heme iron intake and diabetes, CVD, and all-cause mortality among men and women with diabetes in the U.S. population.

**Methods:**

A total of 4416 adults with diabetes(2415 men and 2001 women) from the NHANES 2003–2014 were enrolled. Dietary information was collected by 24-h dietary recall during two nonconsecutive days. Dietary total iron intake was measured based on the dietary survey. Dietary heme iron intake was calculated based on its proportion in dietary total iron intake from each food. non-heme iron is the difference between total iron and heme iron. Diabetes, CVD, and all-cause mortality status were identified as main outcomes. Cox models and RCS analysis were performed to estimate the hazard ratios and 95%CIs.

**Results:**

For men, the participants with a higher dietary heme iron intake were associated with higher risks of CVD (HR_heme iron_ = 1.61,95%CI:1.03–2.51) and all-cause mortality (HR_heme iron_ = 1.42,95%CI:1.10–1.83). For women, participants in the highest quartile of dietary total/heme/non-heme iron intake had a higher diabetes mortality risk ((HR_total iron_ = 2.33,95%CI:1.24–4.38; HR_heme iron_ = 1.87,95%CI:1.00–3.49; HR_non-heme iron_ = 2.28,95%CI:1.19–4.39), compared to those in the lowest quartile. Additionally, the dose–response curve for the relationship between dietary non-heme iron intake and CVD mortality followed a reverse J-shape in women with diabetes.

**Conclusions:**

Higher dietary heme iron intake was associated with an increased CVD mortality risk in both men and women with diabetes. Additionally, higher dietary total, heme, and non-heme iron intake was linked to an increased risk of diabetes mortality among women with diabetes. Therefore, women with diabetes should pay more attention on the overconsumption of any type of dietary iron.

## Funding disclosure

This work was supported by 10.13039/100014717National Natural Science Foundation (32071036, 82101409, 31900697), Heilongjiang Province Natural Science Foundation key project(ZD2022H001) and Scientific Research And Innovation Fund of the First Affiliated Hospital of 10.13039/501100012593Harbin Medical University (2021M24).

## 1 Introduction

The worldwide prevalence of diabetes has tripled in the past few decades, with an estimated 451 million adults living with type 2 diabetes in 2017 [1]. Deaths from diabetes and its related complications have become major threats to public health [2]. Effective dietary factors are warranted to be identified for preventing the progression of diabetes [3]. Iron, primarily obtained from the diet in the form of heme or non-heme iron, is a crucial mineral for human beings, since it widely participates in oxygen transport and basic metabolic processes [4,5]. Previous evidence has demonstrated that iron metabolism disorders coexist with a variety of chronic metabolic diseases, such as diabetes and its major complications (cardiovascular diseases, CVD) [6,7]. However, limited research has focused on the relationship between different types of dietary iron intake and mortality risks of diabetes, CVD, and all-cause among diabetes.

A case–control research indicated that pathological iron overload played a direct role in the pathogenesis of β-cell failure and insulin secretion in diabetes [8]. In-vitro studies also demonstrated that iron overload could induce insulin resistance through the reduction of adiponectin and the inhibition of insulin receptors [9,10]. Additionally, evidence also showed that the hydroxyl free radicals induced by the overaccumulation of iron could increase the oxidation of low-density lipoproteins, thus resulting in cell necrosis, apoptosis, atherosclerosis, and vascular endothelial damage [11,12]. Moreover, emerging epidemiological studies suggested that dietary heme iron intake was associated with higher risks of diabetes, cardiovascular related events and mortality in both men and women [13].

However, several studies have observed that dietary total iron and non-heme iron intake were not associated with any CVD endpoints in either men or women [[14], [15], [16]]. A recent study involving a Chinese population reported that dietary total iron and non-heme iron intake had an L-shaped relationship with diabetes incidence risk in women and an inverse J-shaped relationship in men [17], indicating different relationships existed in different sexes. Additionally, the high absorption rate of heme iron and the inherently higher iron stores have consistently considered to be the main reasons for diabetes and its complication risks, particularly among men [18]. Hence, under this context, high-quality prospective cohort studies were necessary to illustrate which dietary iron types are associated with diabetes-related mortality in men and women with diabetes, enhancing our understanding of the role of sex in these relationships.

Therefore, the present study aims to prospectively examine the associations of dietary total iron, heme, and non-heme iron intake with CVD, diabetes, and all-cause mortality risks among diabetes with different sex using the NHANES database, providing more evidence for therapeutic strategies in diabetes management.

## 2 Method

### 2.1 Study population

**Fig. 1:**
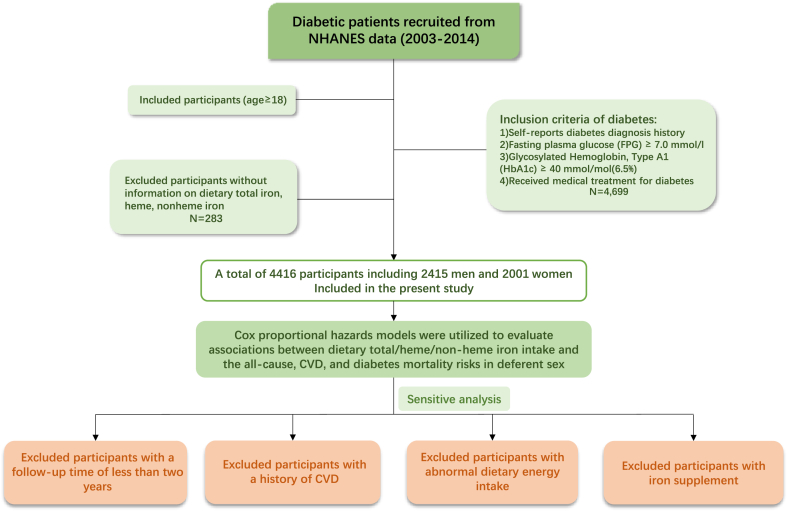
The detailed flowchart of this study.

The dietary and health data utilized in this study were sourced from NHANES(2003–2014). NHANES employs a multi-stage, stratified probability sampling design, allowing for the examination of health and nutritional status across a representative cohort of the U.S. population. This study included participants aged 18 years or older who were diagnosed with diabetes, defined by either self-reported diagnosis, fasting plasma glucose (FPG) levels≥7.0 mmol/l, Glycosylated Hemoglobin (HbA1c)≥40 mmol/mol(6.5 %), or receipt of medical treatment for diabetes, as per established criteria [14]. Individuals with incomplete dietary data on total iron, heme iron, and non-heme iron intake were excluded. Ultimately, the final analysis comprised 4416 participants(2415 men and 2001 women) with complete covariate information. The study flowchart is presented in Fig. 1. Approval for the study was granted by the Research Ethics Review Board of the National Center for Health Statistics, and all participants provided written informed consent.

### 2.2 Main exposures

The intake of dietary total iron, heme iron, and non-heme iron was evaluated through two non-consecutive 24-h dietary recall surveys, a method that has been comprehensively described in previous literature [19,20]. To estimate the average intake of these iron forms, the USDA Food and Nutrient Database for Dietary Studies (FNDDS) was utilized. As the proportion of dietary heme iron contributing to total iron availability varies across different meat types, heme iron intake was calculated by assigning 65 % to beef, 39 % to pork, 52 % to other red meats and processed meats, 26 % to fish and poultry, and 21 % to offal [19]. Non-heme iron intake was determined using the following formula: dietary total iron intake (mg/day) minus dietary heme iron intake (mg/day) [19].

### 2.3 Main outcomes

The primary outcomes of this study were mortality due to CVD, diabetes, and all-cause mortality. Mortality data were obtained from the National Death Index (NDI) through December 31, 2015, using a probabilistic matching algorithm to verify the death status of NHANES participants. Cause-specific mortality was classified according to the 10th Revision of the International Classification of Diseases(ICD-10), with CVD mortality defined by codes I00-I09, I11, I13, I20-I51, or I60-I69, and diabetes mortality by codes E10-E14. Over a follow-up period encompassing 26,101 person-years, a total of 857 participants(519 men, 338 women) died from all causes, 300 participants(182 men, 118 women) succumbed to CVD, and 253 participants(155 men, 98 women) died from diabetes.

### 2.4 Measurements of covariates

Potential covariates considered in the analysis included age (years), engagement in regular physical exercise (yes/no), race (non-Hispanic white, non-Hispanic black, Mexican American, other), educational attainment (less than 9th grade, 9th-11th grade, high school diploma or equivalent, some college or Associate's degree, or bachelor's degree or higher), annual household income (less than $20,000, $20,000-$45,000, $45,000-$75,000, $75,000-$100,000, or greater than $100,000), current smoking status (yes/no), current alcohol consumption (yes/no), body mass index (BMI, kg/m^2^), presence of hypertension and cardiovascular disease (yes/no), use of medication for managing dyslipidemia, hypertension, or diabetes (yes/no), systolic blood pressure (SBP, mmHg), diastolic blood pressure (DBP, mmHg), fasting plasma glucose (FPG, mmol/L), high-density lipoprotein cholesterol (HDL-c, mmol/L), low-density lipoprotein cholesterol (LDL-c, mmol/L), total cholesterol (TC, mmol/L), alanine transaminase (ALT, U/L), aspartate transaminase (AST, U/L), and fasting serum insulin levels. Additionally, daily dietary intake was assessed, including energy (kcal/day), fatty acids (g/day), protein (g/day), cholesterol (mg/day), carbohydrates (g/day), fiber (g/day), and iron supplementation (yes/no). The Alternative Healthy Eating Index (AHEI) was computed to assess the overall quality of the diet.

### 2.5 Statistical analysis

The baseline characteristics of participants encompassed demographic information, dietary intake details, and biochemical measurements. Continuous variables were summarized using mean and standard deviation, while categorical variables were expressed as percentages. Differences in total dietary iron, heme iron, and non-heme iron across sex were examined using a general linear model for continuous variables and a chi-square test for categorical variables.

To assess the associations between dietary total iron, heme iron, and non-heme iron intake with all-cause mortality, CVD mortality, and diabetes mortality, Cox proportional hazards models were employed. Survival time was defined as the interval in person-months from the NHANES interview date to either death or censoring as of December 31, 2015. To more precisely characterize the relationship between dietary iron intake (total, heme, and non-heme) and the risks of all-cause and cause-specific mortality, restricted cubic spline analysis was conducted with knots placed at the 5th, 25th, 75th, and 95th percentiles, to explore both linear and non-linear associations. Additionally, Pearson correlation analysis was performed to evaluate the relationships between various types of dietary iron intake and indicators relevant to the pathological process of diabetes, including FPG, fasting serum insulin, glycated hemoglobin, HDL-c, LDL-c, triglycerides, ALT, AST, and dietary energy intake. Important potential confounders were controlled for in both Cox proportional hazards models and restricted cubic spline analysis. Statistical significance was set at a two-sided p-value of less than 0.05, and analyses were conducted using R version 4.0.2.

### 2.6 Sensitivity analysis

Four sensitivity analyses were performed to evaluate the robustness of the primary findings in this study. In the first set, participants with a follow-up period of less than two years were excluded to minimize the potential influence of severe acute illnesses or accidents. In the second set, participants with a history of cardiovascular disease were excluded, and the main analyses for these associations were re-examined. In the third set, the main analyses were repeated after excluding participants whose daily energy intake was outside the range of 450–5000 kcal. In the fourth set, participants who used iron supplements were excluded from the main analyses.

## 3 Results

### 3.1 Baseline characteristics

Total 4416 participants with diabetes were included in the study, comprising 2415 men and 2001 women. At baseline, men with higher intake of dietary heme iron tended to be younger, non-Hispanic white, and have higher Alternative Healthy Eating Index scores, as well as a lower rate of hypertension medication use. Similarly, men with higher intake of dietary non-heme iron were generally younger, non-Hispanic white, and had higher education, physical activity levels, income, dietary energy intake, and AHEI scores (all p-values <0.05, Table 1). Women with higher intake of dietary heme iron exhibited higher BMI, dietary energy intake, and AHEI scores, and were more likely to consume alcohol. Women with higher intake of non-heme iron were also younger, had higher education, family income, dietary energy intake, AHEI scores, and were more likely to use iron supplements (all p-values <0.05, Table 1).

**Table 1:** Baseline characteristics of diabetes according to quartiles of dietary total iron, heme iron, and non-heme iron intake.

Characteristics	Total iron intake					Heme iron intake					Non-heme iron intake				
	Q1	Q2	Q3	Q4	P-value	Q1	Q2	Q3	Q4	P-value	Q1	Q2	Q3	Q4	P-value
**Men**															
Median intake (mg/d)	8.18	12.25	16.62	24.11		0.26	0.77	1.63	9.00		7.38	11.13	15.22	22.43	
Age (years)	63.1 (13.2)	61.7 (13.7)	60.2 (13.5)	59.5 (14.3)	<0.001	63.2 (13.2)	60.3 (13.7)	61.3 (13.7)	59.6 (14.1)	0.010	62.8 (13.2)	62.2 (13.3)	59.7 (13.9)	59.7 (14.4)	<0.001
Non-Hispanic white [N, (%)]	201 (32.5)	231 (38.6)	260 (43.4)	294 (49.1)	<0.001	221 (36.4)	237 (38.7)	277 (45.9)	251 (42.3)	<0.001	197 (32.3)	239 (39.7)	253 (42.0)	297 (49.3)	<0.001
BMI (kg/m^2^)	31.2 (6.3)	31.0 (6.3)	31.4 (7.0)	31.5 (7.1)	0.327	30.6 (6.4)	31.7 (6.9)	31.6 (6.4)	31.2 (6.8)	0.196	31.3 (6.3)	31.2 (6.3)	31.2 (7.0)	31.5 (7.0)	0.204
Current smoking [N, (%)]	155 (25.0)	134 (22.4)	125 (20.9)	140 (23.4)	0.219	127 (20.9)	152 (24.8)	134 (22.2)	141 (23.8)	0.384	154 (25.3)	139 (23.1)	131 (21.8)	130 (21.6)	0.291
Current drinking [N, (%)]	456 (73.7)	431 (72.1)	466 (77.8)	451 (75.3)	0.053	446 (73.5)	474 (77.5)	446 (74.0)	438 (73.9)	0.485	443 (72.7)	440 (73.1)	471 (78.2)	450 (74.8)	0.107
Regular exercise [N, (%)]	98 (15.8)	89 (14.9)	113 (18.9)	121 (20.2)	0.100	105 (17.3)	101 (16.5)	115 (19.1)	100 (16.9)	0.528	89 (14.6)	93 (15.4)	113 (18.8)	126 (20.9)	0.037
College graduate or above (%)	191 (30.9)	233 (39.0)	280 (46.7)	296 (49.4)	<0.001	254 (41.8)	234 (38.2)	263 (43.6)	249 (42.0)	0.542	193 (31.7)	233 (38.7)	278 (46.2)	296 (49.2)	<0.001
Annual household income>$100,000 [N, (%)]	33 (5.3)	58 (9.7)	57 (9.5)	56 (9.3)	<0.001	58 (9.6)	48 (7.8)	51 (8.5)	47 (7.9)	0.697	35 (5.7)	53 (8.8)	59 (9.8)	57 (9.5)	<0.001
Prevalence of Hypertension [N, (%)]	402 (64.9)	367 (61.4)	373 (62.3)	369 (61.6)	0.262	385 (63.4)	403 (65.8)	364 (60.4)	359 (60.5)	0.292	402 (66.0)	369 (61.3)	364 (60.5)	376 (62.5)	0.103
Dietary Energy (kcal/d)	1325.6 (427.0)	1846.1 (502.0)	2252.7 (609.4)	2716.8 (943.6)	<0.001	1815.4 (763.0)	1967.6 (779.0)	2175.4 (823.6)	2164.0 (892.6)	0.230	1345.8 (453.0)	1852.4 (523.7)	2253.1 (634.5)	2674.6 (949.4)	<0.001
Dietary Total fat (g/d)	50.8 (21.5)	72.0 (27.3)	89.6 (35.2)	107.7 (46.0)	<0.001	69.3 (36.3)	77.1 (36.5)	88.5 (39.5)	84.4 (43.8)	<0.001	52.2 (22.5)	72.3 (28.5)	89.2 (35.9)	105.8 (46.3)	<0.001
Dietary Protein (g/d)	56.0 (20.4)	77.6 (24.8)	94.2 (30.2)	114.6 (41.5)	<0.001	73.7 (31.8)	83.3 (32.1)	92.9 (34.4)	91.8 (45.5)	<0.001	57.2 (21.8)	78.4 (26.0)	95.0 (32.3)	111.2 (41.6)	<0.001
Dietary Carbohydrate (g/d)	154.5 (63.4)	214.9 (67.3)	259.3 (77.9)	317.7 (119.8)	<0.001	221.5 (99.5)	228.5 (99.8)	242.7 (99.6)	251.6 (114.1)	<0.001	154.8 (64.4)	213.1 (67.9)	260.9 (79.5)	315.9 (118.7)	<0.001
Dietary Fiber (g/d)	10.2 (5.3)	15.7 (6.6)	18.7 (7.4)	24.4 (10.9)	<0.001	16.8 (9.2)	15.7 (8.5)	17.5 (9.1)	18.8 (10.3)	<0.001	10.1 (5.1)	15.4 (6.2)	18.9 (7.6)	24.4 (11.0)	<0.001
AHEI	39.0 (9.5)	50.9 (9.9)	57.7 (11.0)	63.0 (11.3)	<0.001	48.6 (13.5)	51.5 (12.9)	56.1 (13.1)	54.0 (14.6)	<0.001	39.7 (10.2)	50.9 (10.4)	57.4 (11.4)	62.3 (11.5)	<0.001
iron supplement [N, (%)]	57 (9.2)	65 (10.9)	79 (13.2)	85 (14.2)	0.148	84 (13.8)	70 (11.4)	64 (10.6)	68 (11.5)	0.478	53 (8.7)	71 (11.8)	75 (12.5)	87 (14.5)	0.076
**Women**															
Median intake (mg/d)	6.91	9.94	13.26	18.76		0.14	0.39	0.84	1.74		6.14	9.05	12.16	17.88	
Age (years)	62.6 (13.3)	61.5 (13.6)	61.0 (14.0)	59.1 (14.6)	0.131	62.4 (13.6)	60.6 (14.0)	61.8 (13.9)	59.5 (14.1)	0.258	62.1 (13.3)	61.5 (14.0)	61.0 (13.7)	59.8 (14.7)	0.010
Non-Hispanic white [N, (%)]	172 (32.6)	154 (31.4)	179 (36.4)	191 (38.9)	0.299	158 (31.8)	163 (31.3)	187 (38.0)	188 (38.3)	0.004	166 (33.0)	157 (31.5)	178 (35.6)	195 (39.1)	0.289
BMI (kg/m^2^)	32.8 (7.7)	32.8 (7.4)	34.1 (8.8)	34.4 (8.2)	0.292	32.2 (7.5)	33.8 (8.1)	33.7 (8.0)	34.6 (8.5)	0.026	32.8 (7.8)	33.0 (7.5)	33.8 (8.5)	34.6 (8.3)	0.300
Current smoking [N, (%)]	87 (16.5)	74 (15.1)	89 (16.9)	67 (13.6)	0.752	70 (14.1)	84 (16.1)	71 (14.4)	86 (17.5)	0.109	92 (18.3)	69 (13.8)	78 (15.6)	72 (14.4)	0.594
Current drinking [N, (%)]	207 (39.3)	198 (40.3)	233 (47.4)	214 (43.6)	0.159	174 (35.0)	238 (45.7)	230 (46.7)	210 (42.8)	0.001	208 (41.4)	192 (38.5)	232 (46.4)	220 (44.1)	0.290
Regular exercise [N, (%)]	69 (13.1)	71 (14.5)	78 (15.9)	80 (16.3)	0.381	65 (13.1)	84 (16.1)	79 (16.1)	70 (14.3)	0.299	65 (12.9)	69 (13.8)	83 (16.6)	81 (16.2)	0.376
College graduate or above (%)	159 (30.2)	169 (34.4)	184 (37.4)	207 (42.2)	0.002	160 (32.2)	196 (37.6)	185 (37.6)	178 (36.3)	0.551	149 (29.6)	168 (33.7)	192 (38.4)	210 (42.1)	0.002
Annual household income>$100,000 [N, (%)]	17 (3.2)	15 (3.1)	33 (6.7)	22 (4.5)	0.001	24 (4.8)	24 (4.6)	22 (4.5)	17 (3.5)	0.366	15 (3.0)	21 (4.2)	28 (5.6)	23 (4.6)	0.015
Prevalence of Hypertension [N, (%)]	385 (73.1)	331 (67.4)	347 (70.5)	345 (70.3)	0.411	356 (71.6)	361 (69.3)	361 (73.4)	330 (67.2)	0.243	365 (72.6)	332 (66.5)	361 (72.2)	350 (70.1)	0.242
Dietary Energy (kcal/d)	1068.9 (339.5)	1447.4 (360.2)	1744.6 (463.6)	2042.6 (684.1)	<0.001	1383.5 (572.7)	1543.5 (501.3)	1595.6 (561.5)	1748.4 (613.3)	<0.001	1072.3 (340.6)	1429.5 (372.2)	1755.5 (491.1)	2013.7 (673.6)	<0.001
Dietary Total fat (g/d)	39.8 (18.1)	55.5 (20.8)	68.9 (26.7)	78.6 (36.3)	<0.001	50.7 (27.5)	59.4 (30.3)	62.0 (27.6)	69.3 (31.9)	0.001	40.2 (18.0)	54.9 (21.1)	69.2 (28.2)	77.1 (35.6)	<0.001
Dietary Protein (g/d)	45.4 (15.6)	61.3 (17.4)	71.8 (20.8)	85.1 (30.0)	<0.001	55.5 (22.8)	62.6 (24.5)	67.4 (24.2)	77.1 (28.0)	<0.001	46.2 (16.3)	60.7 (18.6)	72.7 (22.8)	82.8 (29.1)	<0.001
Dietary Carbohydrate (g/d)	133.3 (50.0)	178.1 (53.0)	212.0 (60.8)	253.2 (89.9)	<0.001	180.0 (76.8)	191.6 (78.2)	195.0 (77.7)	205.9 (80.3)	0.179	132.2 (50.0)	175.8 (53.3)	212.9 (62.0)	251.8 (88.3)	<0.001
Dietary Fiber (g/d)	9.4 (4.4)	12.7 (4.6)	15.5 (6.0)	19.3 (8.0)	<0.001	14.1 (7.3)	13.7 (6.7)	14.1 (7.1)	14.7 (6.6)	0.001	9.2 (4.2)	12.6 (4.6)	15.5 (6.0)	19.3 (8.0)	<0.001
AHEI	35.3 (8.7)	44.2 (9.0)	50.5 (10.7)	54.9 (12.2)	<0.001	41.8 (12.4)	45.1 (12.3)	47.1 (11.8)	50.3 (12.4)	<0.001	35.2 (8.6)	44.1 (9.3)	50.6 (10.9)	54.3 (12.3)	<0.001
iron supplement [N, (%)]	64 (12.1)	65 (13.2)	94 (19.1)	91 (18.5)	0.010	83 (16.7)	77 (14.8)	72 (14.6)	82 (16.7)	0.600	59 (11.7)	66 (13.2)	100 (20.0)	89 (17.8)	0.005

Continuous data are presented as mean (SD, standard deviation). Categorical data are presented as number (%, percentage).

P values were calculate by using general linear model for continuous variables and chi-squared test for categorical variables, respectively.

BMI, body mass index; CVD, cardiovascular disease; SBP, systolic blood pressure; DBP, diastolic blood pressure; FPG, fasting plasma glucose; TC, total cholesterol; HDL-c, high density cholesterol lipoprotein; LDL-c, low density cholesterol lipoprotein; AST, Aspartate transaminase; ALT, Glutamic pyruvic transaminase; AHEI, Alternative Healthy Eating Index.

In both men and women, higher quartiles of dietary heme and non-heme iron intake were associated with increased consumption of total fat, protein, carbohydrate, and dietary fiber, but not dietary cholesterol (all p-values <0.05, Table 1). No significant differences were observed in other variables across quartiles of dietary total, heme, or non-heme iron intake.

### 3.2 Association analysis of dietary total/heme/non-heme iron intake with all-cause, CVD, and diabetes mortality

The associations between dietary total iron, heme iron, and non-heme iron intake and the risks of all-cause, CVD and diabetes mortality among men and women with diabetes are detailed in Table 2, Table 3. Among men, dietary total iron and non-heme iron intake were associated with reduced risks of CVD, diabetes, and all-cause mortality in initial models adjusting for demographic, sociological, and disease-related factors (Models 1 and 2). However, after further adjustment for dietary factors (Model 3), these associations became non-significant, suggesting that the observed protective effects were attributable to the dietary factors. In contrast, men in the highest quartile of dietary heme iron intake exhibited significantly increased risks of CVD (HR_heme iron_ = 1.61, 95%CI:1.03–2.51) and all-cause mortality (HR_heme iron_ = 1.42, 95%CI:1.10–1.83), compared to those in the lowest quartile, even after accounting for dietary factors.

**Table 2:** HR and 95%CI for the association of dietary total iron, heme iron, and non-heme iron intake with all-cause and disease-specific mortality among men with diabetes.

Subgroup	Cases/N	Model 1		Model 2		Model 3	
		HR(95%CI)	P	HR(95%CI)	P	HR(95%CI)	P
**All-cause**							
Dietary iron							
Q1	160/619	Ref	0.015	Ref	0.022	Ref	0.292
Q2	124/598	0.80(0.63,1.01)		0.80(0.63,1.02)		0.85(0.66,1.10)	
Q3	130/599	0.90(0.71,1.13)		0.93(0.73,1.19)		0.99(0.74,1.31)	
Q4	105/599	0.69(0.54,0.89)		0.70(0.54,0.90)		0.79(0.57,1.09)	
Dietary heme iron							
Q1	116/607	Ref	0.009	Ref	0.024	Ref	0.014
Q2	132/612	1.26(0.98,1.62)		1.27(0.99,1.64)		1.24(0.96,1.60)	
Q3	135/603	1.25(0.97,1.60)		1.15(0.89,1.48)		1.20(0.92,1.55)	
Q4	136/593	1.42(1.11,1.82)		1.40(1.09,1.80)		1.42(1.10,1.83)	
Dietary non-heme iron							
Q1	154/609	Ref	0.005	Ref	0.012	Ref	0.143
Q2	138/602	0.86(0.69,1.09)		0.89(0.70,1.12)		0.92(0.72,1.18)	
Q3	119/602	0.82(0.65,1.05)		0.87(0.68,1.11)		0.89(0.67,1.17)	
Q4	108/602	0.70(0.54,0.90)		0.72(0.56,0.92)		0.79(0.57,1.08)	
**CVD mortality**							
Dietary iron							
Q1	62/619	Ref	0.028	Ref	0.062	Ref	0.156
Q2	44/598	0.74(0.50,1.09)		0.73(0.49,1.08)		0.75(0.49,1.14)	
Q3	42/599	0.77(0.52,1.14)		0.85(0.56,1.28)		0.86(0.53,1.39)	
Q4	34/599	0.61(0.40,0.93)		0.63(0.41,0.96)		0.62(0.36,1.09)	
Dietary heme iron							
Q1	35/607	Ref	0.034	Ref	0.067	Ref	0.050
Q2	49/612	1.48(0.96,2.29)		1.47(0.95,2.29)		1.46(0.94,2.29)	
Q3	50/603	1.54(1.00,2.37)		1.38(0.89,2.15)		1.48(0.95,2.32)	
Q4	48/593	1.63(1.05,2.53)		1.58(1.02,2.46)		1.61(1.03,2.51)	
Dietary non-heme iron							
Q1	59/609	Ref	0.021	Ref	0.057	Ref	0.130
Q2	45/602	0.74(0.50,1.09)		0.76(0.51,1.13)		0.78(0.51,1.18)	
Q3	47/602	0.87(0.59,1.28)		0.95(0.64,1.42)		0.95(0.61,1.50)	
Q4	31/602	0.55(0.35,0.85)		0.59(0.38,0.91)		0.58(0.33,1.01)	
**Diabetes mortality**							
Dietary iron							
Q1	56/619	Ref	0.013	Ref	0.018	Ref	0.214
Q2	35/598	0.67(0.44,1.02)		0.70(0.45,1.08)		0.74(0.47,1.18)	
Q3	39/599	0.79(0.52,1.19)		0.83(0.54,1.27)		0.91(0.54,1.54)	
Q4	25/599	0.51(0.31,0.82)		0.51(0.32,0.84)		0.61(0.32,1.14)	
Dietary heme iron							
Q1	37/607	Ref	0.629	Ref	0.790	Ref	0.646
Q2	42/612	1.22(0.79,1.91)		1.24(0.79,1.95)		1.22(0.77,1.92)	
Q3	39/603	1.13(0.72,1.78)		1.05(0.67,1.65)		1.13(0.71,1.81)	
Q4	37/593	1.16(0.73,1.83)		1.13(0.71,1.80)		1.15(0.72,1.84)	
Dietary non-heme iron							
Q1	51/609	Ref	0.044	Ref	0.065	Ref	0.575
Q2	38/602	0.74(0.49,1.13)		0.77(0.50,1.17)		0.83(0.53,1.31)	
Q3	39/602	0.83(0.55,1.27)		0.89(0.58,1.37)		1.02(0.62,1.69)	
Q4	27/602	0.58(0.36,0.93)		0.59(0.37,0.95)		0.77(0.42,1.40)	

Data are HRs and 95 % CI; P values were calculated by using multivariate Cox proportional hazards models.

Model 1 adjustment for age, sport, race, education, income.

Model 2 additionally adjustment for BMI, smoke, drink, SBP, DBP, prevalence of hypertension and CVD, serum TC, FPG, fasting insulin, AST, ALT, HDL-c, LDL-c, medication for diabetes, hypertension, and dyslipidemia.

Model 3 additionally adjustment for dietary consumption of energy, fiber, fat, protein, carbohydrate, and cholesterol, iron supplement, AHEI.

**Table 3:** HR and 95%CI for the association of dietary total iron, heme iron, and non-heme iron intake with all-cause and disease-specific mortality among women with diabetes.

Subgroup	Cases/N	Model 1		Model 2		Model 3	
		HR(95%CI)	P	HR(95%CI)	P	HR(95%CI)	P
**All-cause**							
Dietary iron							
Q1	101/527	Ref	0.904	Ref	0.849	Ref	0.175
Q2	81/491	0.88(0.66,1.18)		0.84(0.62,1.13)		0.96(0.70,1.31)	
Q3	70/492	0.78(0.57,1.06)		0.80(0.59,1.09)		0.96(0.69,1.35)	
Q4	86/491	1.03(0.77,1.38)		1.07(0.79,1.43)		1.31(0.92,1.85)	
Dietary heme iron							
Q1	93/497	Ref	0.330	Ref	0.390	Ref	0.864
Q2	89/521	0.87(0.65,1.17)		0.87(0.65,1.17)		0.89(0.66,1.19)	
Q3	74/492	0.73(0.54,0.99)		0.77(0.56,1.05)		0.81(0.59,1.11)	
Q4	82/491	0.91(0.68,1.23)		0.91(0.67,1.23)		1.01(0.74,1.39)	
Dietary non-heme iron							
Q1	91/503	Ref	0.959	Ref	0.708	Ref	0.116
Q2	82/499	0.85(0.63,1.15)		0.78(0.58,1.06)		0.89(0.65,1.21)	
Q3	78/500	0.83(0.61,1.12)		0.81(0.60,1.11)		1.00(0.71,1.40)	
Q4	87/499	1.02(0.76,1.37)		1.06(0.79,1.43)		1.32(0.92,1.88)	
**CVD mortality**							
Dietary iron							
Q1	39/527	Ref	0.681	Ref	0.855	Ref	0.603
Q2	23/491	0.67(0.40,1.12)		0.66(0.39,1.11)		0.75(0.43,1.29)	
Q3	28/492	0.81(0.50,1.33)		0.88(0.53,1.45)		1.04(0.60,1.82)	
Q4	28/491	0.88(0.54,1.43)		1.01(0.62,1.67)		1.11(0.61,2.01)	
Dietary heme iron							
Q1	31/497	Ref	0.386	Ref	0.440	Ref	0.661
Q2	36/521	1.05(0.65,1.69)		1.07(0.65,1.75)		1.08(0.66,1.76)	
Q3	24/492	0.70(0.41,1.20)		0.76(0.44,1.31)		0.79(0.46,1.36)	
Q4	27/491	0.90(0.54,1.52)		0.91(0.53,1.54)		0.98(0.57,1.70)	
Dietary non-heme iron							
Q1	33/503	Ref	0.530	Ref	0.978	Ref	0.791
Q2	30/499	0.86(0.52,1.41)		0.76(0.46,1.26)		0.84(0.50,1.42)	
Q3	28/500	0.81(0.48,1.34)		0.83(0.50,1.39)		0.99(0.55,1.77)	
Q4	27/499	0.86(0.52,1.44)		0.99(0.59,1.66)		1.05(0.56,1.95)	
**Diabetes mortality**							
Dietary iron							
Q1	23/527	Ref	0.048	Ref	0.025	Ref	0.004
Q2	20/491	0.93(0.51,1.70)		0.84(0.46,1.54)		0.94(0.50,1.75)	
Q3	23/492	1.10(0.61,1.96)		1.18(0.66,2.12)		1.50(0.80,2.81)	
Q4	32/491	1.68(0.98,2.89)		1.75(1.01,3.02)		2.33(1.24,4.38)	
Dietary heme iron							
Q1	17/497	Ref	0.063	Ref	0.062	Ref	0.063
Q2	26/521	1.36(0.74,2.51)		1.38(0.74,2.56)		1.41(0.76,2.63)	
Q3	24/492	1.27(0.68,2.37)		1.37(0.73,2.58)		1.41(0.75,2.67)	
Q4	31/491	1.83(1.01,3.32)		1.82(1.00,3.33)		1.87(1.00,3.49)	
Dietary non-heme iron							
Q1	21/503	Ref	0.095	Ref	0.036	Ref	0.005
Q2	21/499	0.93(0.51,1.71)		0.83(0.45,1.53)		0.94(0.50,1.78)	
Q3	26/500	1.18(0.66,2.12)		1.24(0.69,2.23)		1.60(0.85,3.02)	
Q4	30/499	1.52(0.86,2.67)		1.65(0.94,2.92)		2.28(1.19,4.39)	

Data are HRs and 95 % CI; P values were calculated by using multivariate Cox proportional hazards models.

Model 1 adjustment for age, sport, race, education, income.

Model 2 additionally adjustment for BMI, smoke, drink, SBP, DBP, prevalence of hypertension and CVD, serum TC, FPG, fasting insulin, AST, ALT, HDL-c, LDL-c, medication for diabetes, hypertension, and dyslipidemia.

Model 3 additionally adjustment for dietary consumption of energy, fiber, fat, protein, carbohydrate, and cholesterol, iron supplement, AHEI.

For women, those in the highest quartiles of dietary total iron, heme iron, and non-heme iron intake had elevated risks of diabetes mortality (HR_total iron_ = 2.33, 95%CI: 1.24–4.38; HR_heme iron_ = 1.87, 95%CI:1.00–3.49; HR_non-heme iron_ = 2.28, 95%CI:1.19–4.39) compared to those in the lowest quartiles. These associations remained significant across all models (Models 1–3).

### 3.3 RCS analysis

**Fig. 2:**
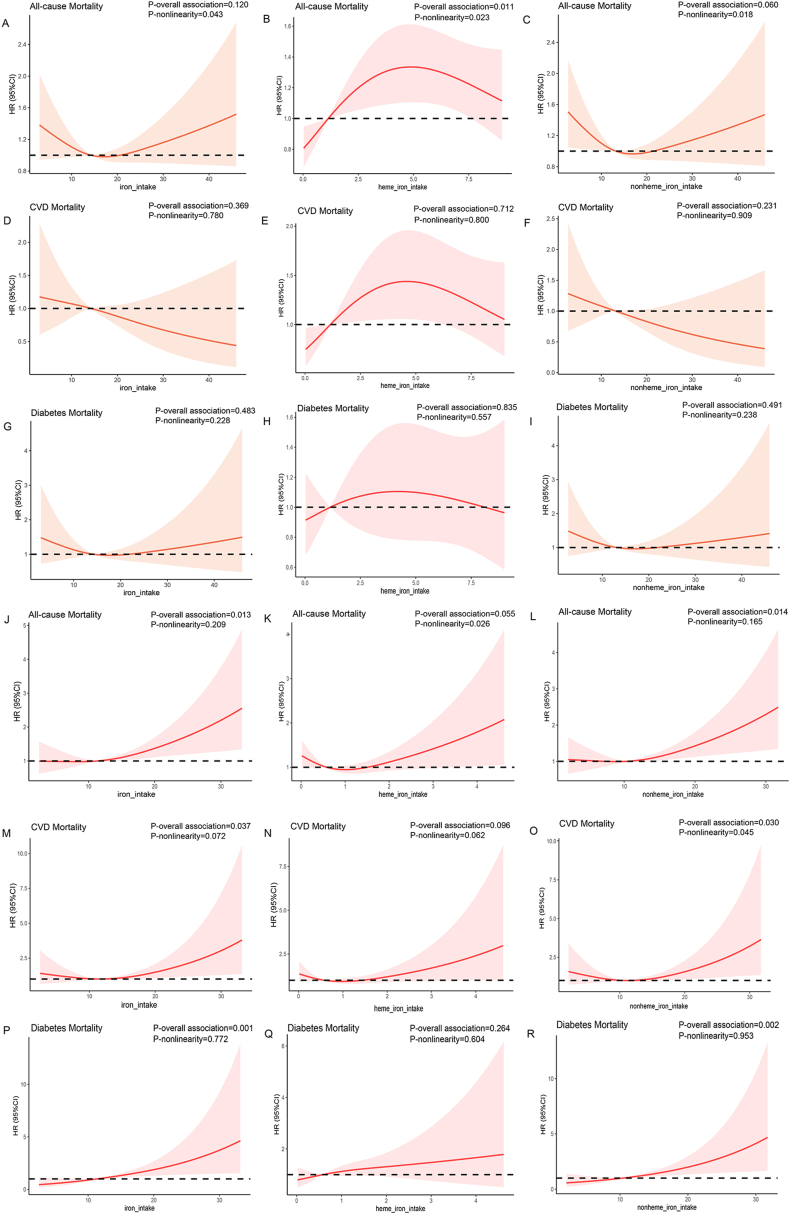
Multivariable-adjusted HRs and 95 % CIs for all-cause mortality according to dietary intake of total (A and J), heme (B and K), and non-heme (C and L) iron in men and women; for CVD mortality according to dietary intake of total (D and M), heme (E and N), and non-heme (F and O) iron in men and women; for diabetes mortality according to dietary intake of total (G and P), heme (H and Q), and non-heme (I and R) iron in men and women, respectively.

The RCS analysis showed that among men with diabetes, a significant nonlinear association between dietary heme iron intake (inverted U) and non-heme iron intake(U Shape) with all-cause mortality(Fig. 2A–C), rather than CVD(Fig. 2D–F) and diabetes mortality(Fig. 2G–I). Among women with diabetes, dietary total/non-heme iron intake showed a significant positive graded relationship with all-cause(Fig. 2J–L) and diabetes mortality(Fig. 2P–R), although these associations were not nonlinear. A reverse J-shaped association was observed between dietary non-heme iron intake and CVD mortality (P-overall association = 0.030, P-nonlinearity = 0.045), rather than total/heme iron intake(Fig. 2M and N). This indicated that when non-heme iron intake is relatively high, there was a positive association with the risk of CVD mortality. The RCS curves also showed that when non-heme iron intake exceeded 11.64 mg/day, the HR was significantly increased (Fig. 2O). Additionally, there was a marginally significant nonlinear association between dietary heme iron intake and all-cause mortality (P-overall association = 0.055, P-nonlinearity = 0.026).

### 3.4 Association analysis of dietary total/heme/non-heme iron intake with diabetes-related indicators

**Fig. 3:**
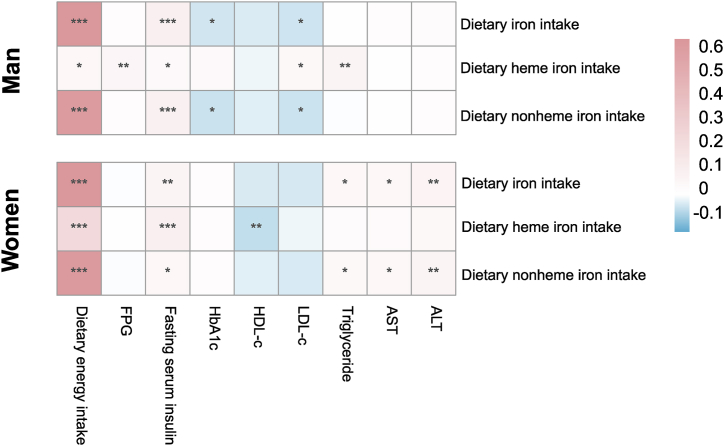
Association analysis of dietary total iron, heme iron, non-heme iron intake with diabetes-related indicators.

The associations of dietary total/heme/non-heme iron intake with diabetes-related indicators are presented in Fig. 3. The positive relationships of dietary total, heme, and non-heme iron intake with dietary energy intake and fasting serum insulin were observed in both sexes. In men with diabetes, dietary heme iron intake was positively associated with FPG (r = 0.059,p = 0.004), LDL-c (r = 0.046,p = 0.024), and Triglyceride levels (r = 0.065,p = 0.001), but dietary total/non-heme iron intake was negatively associated with HbA1c (r = −0.043,p = 0.035; r = −0.048,p = 0.017) and LDL-c levels (r = −0.044, p = 0.032; r = −0.047, p = 0.021).

Among women with diabetes, dietary heme iron intake was negatively associated with HDL-c(r = −0.062,p = 0.006), and dietary total/non-heme iron intake was positively associated with Triglyceride, ALT(r = 0.068,p = 0.002; r = 0.063,p = 0.005) and AST levels (r = 0.054,p = 0.015; r = 0.050, p = 0.025).

### 3.5 Sensitivity analysis

In the first and second sets of sensitivity analyses, the significant associations between dietary heme iron intake and CVD, all-cause mortality among men with diabetes were maintained. Conversely, the associations between dietary total and non-heme iron intake and diabetes and all-cause mortality among women with diabetes were attenuated, indicating robustness in the main analysis results (Supplementary Tables 1–2). In the third set, which focused on participants with energy intake between 450 and 5000 kcal per day, the significant associations for men with diabetes persisted. Additionally, associations between dietary total, non-heme, and heme iron intake and diabetes mortality risk remained for women with diabetes (Supplementary Table 3). In the fourth set, excluding participants with iron supplementation did not substantially alter the correlations observed in the main analyses (Supplementary Table 4).

## 4 Discussion

In this study, we investigated the relationships between dietary total iron, heme iron, and non-heme iron intake and the risks of diabetes, CVD and all-cause mortality among individuals with diabetes, differentiated by sex. Among men with diabetes, after adjusting for traditional risk factors and dietary factors, we observed significant associations between dietary heme iron intake and increased risks of CVD and all-cause mortality. In contrast, dietary non-heme iron intake was not significantly associated with these outcomes. For women with diabetes, higher intake of dietary heme iron was linked to increased diabetes mortality. Furthermore, higher intake of dietary non-heme iron was associated with elevated risks of both diabetes and CVD, following a J-shaped pattern. These findings suggest that individuals with diabetes should carefully manage their dietary iron intake to mitigate the progression of diabetes and associated complications.

The primary finding of this study was the positive association between dietary heme iron intake and CVD mortality among men and women with diabetes. Most prospective studies have indicated that high dietary heme iron intake is linked to significantly increased incidence of diabetes and cardiovascular outcomes [15,17,18,20]. Excessive heme iron intake, due to its high absorption rate, may contribute to the development of CVD in individuals with diabetes. Our study, consistent with previous research, identified a significant association between dietary heme iron intake and CVD mortality among men with diabetes, who had a mean intake of 2.28 mg/day. Dyslipidemia may be a contributing factor, as heme iron intake was positively correlated with LDL-c and triglyceride levels in these men. However, recent studies have suggested that dietary heme iron intake may not be associated with diabetes risk [16]. Two population-based cohort studies conducted in Chinese and Japanese adults found no association between heme iron intake and diabetes incidence, potentially due to the lower levels of heme iron intake in these populations (median intake of 0.75 mg/day for Chinese men, 0.63 mg/day for Chinese women, and mean intake of 0.2 mg/day for Japanese individuals) [21,22]. These lower intakes may be insufficient to influence diabetes risk and related complications.

Despite the median dietary heme iron intake among women with diabetes in this study (0.58 mg/day) being lower than in other studies, we still observed significant associations between dietary heme iron intake and both CVD and diabetes mortality. This suggests that even at relatively lower intake levels, excessive heme iron consumption could lead to adverse outcomes in women with diabetes. Individuals with diabetes are known to be prone to iron metabolism disorders, often resulting in increased internal iron stores [23]. These disorders may lower the threshold at which dietary heme iron becomes harmful, thereby increasing the risk of diabetes mortality. Our study also found a negative correlation between dietary heme iron intake and HDL-c levels, which is a protective factor against elevated blood lipid levels in women with diabetes. This indicates that dietary heme iron intake might significantly contribute to diabetes-related mortality by influencing lipid metabolism in this population. Overall, these findings support a dose-dependent relationship between dietary heme iron intake and mortality from diabetes and CVD among diabetic patients.

In contrast, the relationship between dietary non-heme iron, as a major source of total dietary iron, and diabetes, CVD, and all-cause mortality has been inconsistent in previous research [24,25]. Our study found no significant correlations between dietary non-heme iron intake and diabetes or CVD mortality among men with diabetes, which is consistent with most studies [19]. Additionally, dietary non-heme iron intake was negatively correlated with HbA1c and LDL-c levels, suggesting that higher non-heme iron intake might reduce diabetes-related mortality by lowering iron storage compared to heme iron. However, our study uniquely found that higher dietary non-heme iron intake among women with diabetes was associated with increased mortality from CVD and diabetes. This discrepancy may be attributed to hormonal fluctuations in women, which men do not experience to the same extent. Several studies involving women from different countries support the positive associations between dietary non-heme iron intake and risks of diabetes and CVD [21,[26], [27], [28], [29]]. Conversely, other prospective studies have reported no association or an inverse relationship between dietary non-heme iron intake and diabetes or CVD mortality in the general population [30,31]. For instance, the Nurses' Health Study found no association between dietary non-heme iron intake and diabetes risk in women around 45 years old [32]. Similarly, a study of American women around 40 years old showed a negative association between dietary non-heme iron intake and diabetes risk [33]. The Japan Collaborative Cohort Study observed no association between dietary non-heme iron intake and CVD mortality in women around 55 years old [21]. These variations might be influenced by hormonal changes, with our study's participants averaging 62 years old—significantly older than those in previous studies. Postmenopausal women experience reduced iron loss, leading to higher iron accumulation. A study found that serum ferritin levels in postmenopausal women are 2–3 times higher than in premenopausal women. Another report from the Nurses' Health Study indicated that postmenopausal women with higher dietary iron intake have increased iron stores [34], a key factor in metabolic syndrome. Animal studies have shown that in ovariectomized, estrogen-deficient rat models, iron accumulates in the liver, kidneys, bones, and insulin tissues [35,36]. Our study also observed that higher dietary non-heme iron intake was associated with elevated levels of AST and ALT in women with diabetes, potentially exacerbating the risk of atherosclerosis by impairing glucose tolerance and increasing serum lipid levels, thus heightening diabetes-related mortality. Therefore, further research is needed to comprehensively examine the impact of different dietary iron sources on disease-specific mortality in various populations, which may provide new therapeutic insights.

This study has several strengths. It utilized a prospective cohort design to explore the associations between dietary total iron, heme iron, and non-heme iron intake and the risks of diabetes, CVD, and all-cause mortality among diabetic individuals. Accurate assessments of different dietary iron sources were conducted using two-step consecutive dietary surveys within a week. Moreover, after adjusting for traditional and dietary factors, the associations between dietary iron sources and risks of diabetes, CVD, and all-cause mortality in men and women with diabetes remained robust. Nevertheless, the study has some limitations. First, the observational nature of the study means that changes in covariates over time and unmeasured confounding factors could not be fully controlled. Therefore, large-scale randomized clinical trials with rigorous control of confounders are needed. Second, information on diabetes types was lacking, although NHANES data primarily involve type 2 diabetes [37]. Further research is required to investigate the associations between different dietary iron sources and outcomes in various diabetes types. Third, the study observed a significant sex difference in the relationship between dietary non-heme iron intake and disease-specific mortality, but the underlying causation and mechanisms remain incompletely understood. More molecular mechanistic studies are needed. Finally, since the results are based on an American population, it is essential to analyze the associations between different dietary iron types and diabetes, CVD, and all-cause mortality in other racial populations.

## 5 Clinical implications

Our findings have significant clinical implications. This study provides a comprehensive assessment of the associations between dietary total iron, heme iron, and non-heme iron intake with long-term survival among patients with diabetes, offering valuable insights for dietary prevention of diabetes-related mortality. We observed that higher dietary heme iron intake was linked to increased risks of CVD and all-cause mortality among men with diabetes. Similarly, in women with diabetes, higher dietary heme iron intake was associated with a higher risk of diabetes mortality, while elevated non-heme iron intake was linked to increased mortality from both CVD and diabetes.

These results highlight the need for diabetic patients to be cautious about excessive dietary iron intake, given its potential adverse effects on disease progression. This information is essential for developing informed dietary guidelines and effective treatment strategies for diabetes management.

## Data availability

The data was obtained from official websites: https://www.cdc.gov/nchs/nhanes/index.htm.

## Ethics declarations

This study was approved by the National Center for Health Statistics Research Ethics Review Board gave its approval and obtained written informed consent from all participants. The detailed Research Ethics Committee information and Informed Consent information can be obtained from following websites (https://www.cdc.gov/nchs/nhanes/irba98.htm; https://www.cdc.gov/nchs/nhanes/genetics/genetic_participants.htm).

## CRediT authorship contribution statement

**Yimin Jin:** Writing – review & editing, Validation, Conceptualization. **Yang Huang:** Writing – review & editing, Validation. **Tongshuai Zhang:** Writing – review & editing, Validation. **Qixu Sun:** Writing – review & editing, Visualization. **Yao Zhang:** Writing – review & editing, Validation. **Peiru Zhang:** Writing – review & editing, Validation, Software, Conceptualization. **Guangyou Wang:** Writing – review & editing, Writing – original draft, Validation. **Jingyu Zhang:** Writing – review & editing, Validation. **Jinrong Wu:** Writing – review & editing, Supervision, Funding acquisition.

## Declaration of competing interest

The authors declare the following financial interests/personal relationships which may be considered as potential competing interests:Yimin Jin1, Yang Huang2, Tongshuai Zhang reports financial support was provided by 10.13039/100014717National Natural Science Foundation. Guangyou Wang, Jingyu Zhang, Jinrong Wu reports financial support was provided by Scientific Research And Innovation Fund of the First Affiliated Hospital of 10.13039/501100012593Harbin Medical University. If there are other authors, they declare that they have no known competing financial interests or personal relationships that could have appeared to influence the work reported in this paper.
