# Supplementary material for: Associations of dietary total, heme, non-heme iron intake with diabetes, CVD, and all-cause mortality in men and women with diabetes
Source: Heliyon. 2024 Oct 1;10(19):e38758. doi: 10.1016/j.heliyon.2024.e38758 (PMC11490858; doi:10.1016/j.heliyon.2024.e38758)
Supplement: Multimedia component 1 [file mmc1.docx]

**Supplement Table 1 HR and 95%CI for the association of dietary total iron, heme iron, and nonheme iron intake with all-cause and disease-specific mortality among diabetes who had a follow-up periods over 2 years**

| Subgroup | Cases/N | Model 1 | | Model 2 | | Model 3 | |
| --- | --- | --- | --- | --- | --- | --- | --- |
|  |  | HR(95%CI) | P | HR(95%CI) | P | HR(95%CI) | P |
| **Men** |  |  |  |  |  |  |  |
| **All-cause** |  |  |  |  |  |  |  |
| Dietary iron |  |  |  |  |  |  |  |
| Q1 | 121/522 | Ref | 0.060 | Ref | 0.065 | Ref | 0.153 |
| Q2 | 107/536 | 0.89(0.69,1.16) |  | 0.90(0.69,1.17) |  | 0.90(0.68,1.19) |  |
| Q3 | 103/531 | 0.93(0.71,1.21) |  | 0.96(0.73,1.27) |  | 0.93(0.67,1.28) |  |
| Q4 | 86/534 | 0.74(0.56,0.98) |  | 0.74(0.55,0.98) |  | 0.74(0.51,1.07) |  |
| Dietary heme iron |  |  |  |  |  |  |  |
| Q1 | 88/533 | Ref | 0.006 | Ref | 0.017 | Ref | 0.009 |
| Q2 | 109/538 | 1.45(1.00,2.09) |  | 1.37(0.94,1.98) |  | 1.41(0.97,2.05) |  |
| Q3 | 110/530 | 1.40(0.97,2.02) |  | 1.28(0.88,1.87) |  | 1.30(0.89,1.90) |  |
| Q4 | 110/522 | 1.69(1.19,2.39) |  | 1.57(1.11,2.23) |  | 1.66(1.16,2.37) |  |
| Dietary nonheme iron |  |  |  |  |  |  |  |
| Q1 | 115/512 | Ref | 0.034 | Ref | 0.056 | Ref | 0.139 |
| Q2 | 119/544 | 0.97(0.76,1.28) |  | 1.02(0.79,1.32) |  | 1.00(0.76,1.33) |  |
| Q3 | 95/531 | 0.86(0.65,1.13) |  | 0.91(0.69,1.20) |  | 0.87(0.63,1.20) |  |
| Q4 | 88/536 | 0.76(0.57,1.01) |  | 0.77(0.58,1.03) |  | 0.79(0.55,1.13) |  |
|  |  |  |  |  |  |  |  |
| **CVD mortality** |  |  |  |  |  |  |  |
| Dietary iron |  |  |  |  |  |  |  |
| Q1 | 48/522 | Ref | 0.150 | Ref | 0.146 | Ref | 0.321 |
| Q2 | 34/536 | 0.73(0.47,1.13) |  | 0.68(0.43,1.06) |  | 0.69(0.42,1.11) |  |
| Q3 | 34/531 | 0.80(0.51,1.25) |  | 0.85(0.54,1.35) |  | 0.87(0.51,1.50) |  |
| Q4 | 30/534 | 0.69(0.43,1.09) |  | 0.65(0.41,1.05) |  | 0.66(0.36,1.23) |  |
| Dietary heme iron |  |  |  |  |  |  |  |
| Q1 | 26/533 | Ref | 0.022 | Ref | 0.053 | Ref | 0.037 |
| Q2 | 40/538 | 1.69(0.85,3.35) |  | 1.52(0.76,3.05) |  | 1.62(0.97,2.69) |  |
| Q3 | 41/530 | 1.94(0.99,3.81) |  | 1.67(0.84,3.31) |  | 1.65(0.99,2.76) |  |
| Q4 | 39/522 | 2.14(1.12,4.09) |  | 1.89(1.05,2.85) |  | 1.78(1.07,2.96) |  |
| Dietary nonheme iron |  |  |  |  |  |  |  |
| Q1 | 44/512 | Ref | 0.152 | Ref | 0.185 | Ref | 0.392 |
| Q2 | 38/544 | 0.82(0.53,1.27) |  | 0.83(0.54,1.28) |  | 0.86(0.53,1.37) |  |
| Q3 | 35/531 | 0.85(0.54,1.33) |  | 0.91(0.58,1.44) |  | 0.94(0.56,1.59) |  |
| Q4 | 29/536 | 0.69(0.43,1.11) |  | 0.69(0.43,1.11) |  | 0.73(0.40,1.33) |  |
|  |  |  |  |  |  |  |  |
| **Diabetes mortality** |  |  |  |  |  |  |  |
| Dietary iron |  |  |  |  |  |  |  |
| Q1 | 50/522 | Ref | 0.005 | Ref | 0.003 | Ref | 0.041 |
| Q2 | 26/536 | 0.54(0.34,0.87) |  | 0.54(0.33,0.87) |  | 0.52(0.31,0.88) |  |
| Q3 | 30/531 | 0.67(0.42,1.06) |  | 0.67(0.42,1.08) |  | 0.67(0.37,1.21) |  |
| Q4 | 20/534 | 0.44(0.26,0.75) |  | 0.41(0.24,0.70) |  | 0.52(0.21,1.00) |  |
| Dietary heme iron |  |  |  |  |  |  |  |
| Q1 | 32/533 | Ref | 0.642 | Ref | 0.547 | Ref | 0.918 |
| Q2 | 37/538 | 1.38(0.75,2.53) |  | 1.22(0.78,1.91) |  | 1.38(0.74,2.58) |  |
| Q3 | 29/530 | 1.08(0.58,2.02) |  | 1.09(0.69,1.73) |  | 1.15(0.61,2.19) |  |
| Q4 | 28/522 | 1.07(0.59,1.93) |  | 1.15(0.73,1.83) |  | 1.15(0.62,2.13) |  |
| Dietary nonheme iron |  |  |  |  |  |  |  |
| Q1 | 44/512 | Ref | 0.028 | Ref | 0.021 | Ref | 0.265 |
| Q2 | 30/544 | 0.66(0.42,1.06) |  | 0.68(0.42,1.08) |  | 0.68(0.41,1.13) |  |
| Q3 | 30/531 | 0.73(0.46,1.17) |  | 0.76(0.47,1.23) |  | 0.82(0.46,1.43) |  |
| Q3 | 22/536 | 0.53(0.32,0.90) |  | 0.51(0.30,0.87) |  | 0.63(0.32,1.23) |  |
|  |  |  |  |  |  |  |  |
| **Women** |  |  |  |  |  |  |  |
| **All-cause** |  |  |  |  |  |  |  |
| Dietary iron |  |  |  |  |  |  |  |
| Q1 | 82/465 | Ref | 0.758 | Ref | 0.975 | Ref | 0.482 |
| Q2 | 60/434 | 0.81(0.58,1.13) |  | 0.74(0.53,1.04) |  | 0.81(0.57,1.16) |  |
| Q3 | 55/435 | 0.79(0.56,1.11) |  | 0.82(0.58,1.16) |  | 0.93(0.63,1.36) |  |
| Q4 | 65/435 | 0.97(0.70,1.35) |  | 0.99(0.71,1,38) |  | 1.14(0.77,1.70) |  |
| Dietary heme iron |  |  |  |  |  |  |  |
| Q1 | 75/442 | Ref | 0.190 | Ref | 0.190 | Ref | 0.360 |
| Q2 | 68/455 | 0.79(0.58,1.08) |  | 0.79(0.58,1.07) |  | 0.80(0.58,1.09) |  |
| Q3 | 59/436 | 0.65(0.46,0.92) |  | 0.70(0.49,0.99) |  | 0.71(0.50,1.02) |  |
| Q4 | 60/436 | 0.93(0.62,1.38) |  | 0.86(0.57,1.29) |  | 0.95(0.62,1.46) |  |
| Dietary nonheme iron |  |  |  |  |  |  |  |
| Q1 | 72/445 | Ref | 0.858 | Ref | 0.910 | Ref | 0.369 |
| Q2 | 63/441 | 0.81(0.58,1.14) |  | 0.75(0.53,1.06) |  | 0.82(0.57,1.17) |  |
| Q3 | 62/442 | 0.84(0.60,1.19) |  | 0.82(0.58,1.16) |  | 0.94(0.64,1.39) |  |
| Q4 | 65/441 | 0.96(0.69,1.35) |  | 1.01(0.72,1.42) |  | 1.18(0.79,1.78) |  |
|  |  |  |  |  |  |  |  |
| **CVD mortality** |  |  |  |  |  |  |  |
| Dietary iron |  |  |  |  |  |  |  |
| Q1 | 32/465 | Ref | 0.753 | Ref | 0.873 | Ref | 0.986 |
| Q2 | 17/434 | 0.61(0.34,1.10) |  | 0.61(0.33,1.11) |  | 0.63(0.34,1.19) |  |
| Q3 | 19/435 | 0.71(0.40,1.27) |  | 0.79(0.441.41) |  | 0.79(0.41,1.52) |  |
| Q4 | 23/435 | 0.92(0.54,1.58) |  | 1.04(0.60,1.79) |  | 0.97(0.50,1.91) |  |
| Dietary heme iron |  |  |  |  |  |  |  |
| Q1 | 24/442 | Ref | 0.422 | Ref | 0.349 | Ref | 0.382 |
| Q2 | 27/455 | 0.94(0.54,1.63) |  | 0.93(0.53,1.63) |  | 0.91(0.52,1.61) |  |
| Q3 | 21/436 | 0.77(0.43,1.38) |  | 0.81(0.45,1.47) |  | 0.80(0.44,1.48) |  |
| Q4 | 19/436 | 0.83(0.46,1.53) |  | 0.77(0.42,1.44) |  | 0.78(0.41,1.48) |  |
| Dietary nonheme iron |  |  |  |  |  |  |  |
| Q1 | 26/445 | Ref | 0.734 | Ref | 0.817 | Ref | 0.981 |
| Q2 | 22/441 | 0.78(0.44,1.38) |  | 0.73(0.41,1.30) |  | 0.72(0.39,1.32) |  |
| Q3 | 21/442 | 0.78(0.43,1.39) |  | 0.81(0.45,1.46) |  | 0.83(0.43,1.62) |  |
| Q4 | 22/441 | 0.91(0.51,1.62) |  | 1.06(0.59,1.90) |  | 0.97(0.47,1.99) |  |
|  |  |  |  |  |  |  |  |
| **Diabetes mortality** |  |  |  |  |  |  |  |
| Dietary iron |  |  |  |  |  |  |  |
| Q1 | 20/465 | Ref | 0.364 | Ref | 0.223 | Ref | 0.140 |
| Q2 | 18/434 | 0.95(0.50,1.80) |  | 0.89(0.46,1.70) |  | 0.86(0.43,1.70) |  |
| Q3 | 17/435 | 0.97(0.51,1.87) |  | 1.08(0.56,2.09) |  | 1.19(0.58,2.42) |  |
| Q4 | 22/435 | 1.34(0.73,2.48) |  | 1.43(0.77,2.65) |  | 1.64(0.79,3.41) |  |
| Dietary heme iron |  |  |  |  |  |  |  |
| Q1 | 15/442 | Ref | 0.336 | Ref | 0.397 | Ref | 0.466 |
| Q2 | 21/455 | 1.31(0.71,2.42) |  | 1.31(0.70,2.45) |  | 1.16(0.59,2.28) |  |
| Q3 | 18/436 | 0.76(0.37,1.57) |  | 0.83(0.40,1.74) |  | 1.02(0.50,2.06) |  |
| Q4 | 23/436 | 1.99(0.98,4.05) |  | 1.79(0.87,3.70) |  | 1.37(0.68,2.75) |  |
| Dietary nonheme iron |  |  |  |  |  |  |  |
| Q1 | 18/445 | Ref | 0.496 | Ref | 0.249 | Ref | 0.129 |
| Q2 | 19/441 | 0.95(0.50,1.82) |  | 0.91(0.47,1.77) |  | 0.91(0.46,1.82) |  |
| Q3 | 19/442 | 1.02(0.53,1.96) |  | 1.09(0.56,2.11) |  | 1.22(0.59,2.50) |  |
| Q3 | 21/441 | 1.23(0.65,2.33) |  | 1.41(0.74,2.71) |  | 1.72(0.80,3.70) |  |

Data are HRs and 95%CI; P values were calculated by using multivariate Cox proportional hazards models;

Model 1 adjustment for age, sport, race, education, income;

Model 2 additionally adjustment for BMI, smoke, drink, SBP, DBP, prevalence of hypertension and CVD, serum TC, FPG, fasting insulin, AST, ALT, HDL, LDL, medication for diabetes, hypertension, and dyslipidemia;

Model 3 additionally adjustment for dietary consumption of energy, fiber, fat, protein, carbohydrate, and cholesterol, iron supplement, AHEI.

**Supplement Table 2 HR and 95%CI for the association of dietary total iron, heme iron, and nonheme iron intake with all-cause and disease-specific mortality among diabetes without a history of CVD**

| Subgroup | Cases/N | Model 1 | | Model 2 | | Model 3 | |
| --- | --- | --- | --- | --- | --- | --- | --- |
|  |  | HR(95%CI) | P | HR(95%CI) | P | HR(95%CI) | P |
| **Men** |  |  |  |  |  |  |  |
| **All-cause** |  |  |  |  |  |  |  |
| Dietary iron |  |  |  |  |  |  |  |
| Q1 | 85/425 | Ref | 0.018 | Ref | 0.020 | Ref | 0.473 |
| Q2 | 63/415 | 0.77(0.55,1.07) |  | 0.81(0.58,1.14) |  | 0.92(0.65,1.32) |  |
| Q3 | 64/445 | 0.79(0.57,1.10) |  | 0.81(0.58,1.14) |  | 0.95(0.64,1.40) |  |
| Q4 | 49/440 | 0.63(0.44,0.91) |  | 0.64(0.44,0.92) |  | 0.82(0.52,1.31) |  |
| Dietary heme iron |  |  |  |  |  |  |  |
| Q1 | 59/431 | Ref | 0.016 | Ref | 0.021 | Ref | 0.004 |
| Q2 | 64/440 | 1.04(0.68,1.58) |  | 0.96(0.63,1.46) |  | 1.01(0.65,1.55) |  |
| Q3 | 61/429 | 1.05(0.69,1.61) |  | 0.98(0.64,1.51) |  | 1.05(0.68,1.63) |  |
| Q4 | 77/425 | 1.47(1.00,2.16) |  | 1.40(0.95,2.07) |  | 1.58(1.06,2.37) |  |
| Dietary nonheme iron |  |  |  |  |  |  |  |
| Q1 | 82/411 | Ref | 0.005 | Ref | 0.007 | Ref | 0.170 |
| Q2 | 68/419 | 0.75(0.54,1.03) |  | 0.78(0.56,1.08) |  | 0.85(0.59,1.20) |  |
| Q3 | 61/453 | 0.71(0.51,1.00) |  | 0.74(0.53,1.04) |  | 0.81(0.54,1.20) |  |
| Q4 | 50/442 | 0.60(0.42,0.86) |  | 0.61(0.42,0.87) |  | 0.72(0.46,1.14) |  |
|  |  |  |  |  |  |  |  |
| **CVD mortality** |  |  |  |  |  |  |  |
| Dietary iron |  |  |  |  |  |  |  |
| Q1 | 29/425 | Ref | 0.024 | Ref | 0.033 | Ref | 0.155 |
| Q2 | 17/415 | 0.63(0.34,1.15) |  | 0.68(0.37,1.25) |  | 0.71(0.37,1.38) |  |
| Q3 | 22/445 | 0.81(0.46,1.43) |  | 0.86(0.48,1.52) |  | 0.93(0.46,1.85) |  |
| Q4 | 9/440 | 0.36(0.17,0.78) |  | 0.38(0.18,0.81) |  | 0.41(0.16,1.05) |  |
| Dietary heme iron |  |  |  |  |  |  |  |
| Q1 | 12/431 | Ref | 0.007 | Ref | 0.005 | Ref | 0.004 |
| Q2 | 18/440 | 2.11(0.78,5.72) |  | 2.13(0.78,5.83) |  | 2.00(0.72,5.50) |  |
| Q3 | 21/429 | 2.18(0.80,5.96) |  | 2.16(0.78,5.93) |  | 2.12(0.76,5.93) |  |
| Q4 | 26/425 | 3.31(1.29,8.50) |  | 3.45(1.33,8.95) |  | 3.48(1.33,9.10) |  |
| Dietary nonheme iron |  |  |  |  |  |  |  |
| Q1 | 26/411 | Ref | 0.027 | Ref | 0.032 | Ref | 0.152 |
| Q2 | 18/419 | 0.64(0.35,1.18) |  | 0.66(0.36,1.21) |  | 0.69(0.36,1.33) |  |
| Q3 | 25/453 | 0.91(0.52,1.59) |  | 0.95(0.54,1.68) |  | 1.03(0.52,2.02) |  |
| Q4 | 8/442 | 0.32(0.14,0.72) |  | 0.32(0.14,0.73) |  | 0.36(0.14,0.94) |  |
|  |  |  |  |  |  |  |  |
| **Diabetes mortality** |  |  |  |  |  |  |  |
| Dietary iron |  |  |  |  |  |  |  |
| Q1 | 31/425 | Ref | 0.066 | Ref | 0.049 | Ref | 0.135 |
| Q2 | 16/415 | 0.57(0.31,1.04) |  | 0.63(0.34,1.17) |  | 0.62(0.32,1.21) |  |
| Q3 | 20/445 | 0.71(0.40,1.25) |  | 0.70(0.39,1.25) |  | 0.69(0.34,1.40) |  |
| Q4 | 13/440 | 0.51(0.26,0.99) |  | 0.50(0.25,0.97) |  | 0.49(0.21,1.15) |  |
| Dietary heme iron |  |  |  |  |  |  |  |
| Q1 | 20/431 | Ref | 0.790 | Ref | 0.787 | Ref | 0.788 |
| Q2 | 22/440 | 1.37(0.67,2.78) |  | 1.22(0.78,1.91) |  | 1.25(0.60,2.60) |  |
| Q3 | 18/429 | 0.81(0.37,1.77) |  | 1.09(0.69,1.73) |  | 0.76(0.34,1.72) |  |
| Q4 | 20/425 | 1.12(0.55,2.27) |  | 1.15(0.73,1.83) |  | 1.07(0.51,2.26) |  |
| Dietary nonheme iron |  |  |  |  |  |  |  |
| Q1 | 27/411 | Ref | 0.155 | Ref | 0.160 | Ref | 0.450 |
| Q2 | 18/419 | 0.63(0.34,1.14) |  | 0.65(0.35,1.20) |  | 0.67(0.34,1.29) |  |
| Q3 | 21/453 | 0.79(0.44,1.40) |  | 0.84(0.47,1.51) |  | 0.89(0.44,1.80) |  |
| Q3 | 14/442 | 0.57(0.29,1.10) |  | 0.56(0.29,1.10) |  | 0.65(0.28,1.49) |  |
|  |  |  |  |  |  |  |  |
| **Women** |  |  |  |  |  |  |  |
| **All-cause** |  |  |  |  |  |  |  |
| Dietary iron |  |  |  |  |  |  |  |
| Q1 | 47/390 | Ref | 0.256 | Ref | 0.599 | Ref | 0.171 |
| Q2 | 40/371 | 0.93(0.61,1.42) |  | 0.90(0.58,1.38) |  | 1.00(0.65,1.66) |  |
| Q3 | 40/372 | 0.90(0.61,1.32) |  | 0.83(0.56,1.23) |  | 0.92(0.60,1.41) |  |
| Q4 | 44/371 | 1.52(0.96,2.39) |  | 1.27(0.79,2.04) |  | 1.36(0.96,3.24) |  |
| Dietary heme iron |  |  |  |  |  |  |  |
| Q1 | 48/376 | Ref | 0.275 | Ref | 0.222 | Ref | 0.265 |
| Q2 | 49/384 | 1.05(0.65,1.71) |  | 0.99(0.58,1.60) |  | 1.01(0.59,1.71) |  |
| Q3 | 34/376 | 0.59(0.38,0.93) |  | 0.62(0.39,0.97) |  | 0.61(0.39,0.97) |  |
| Q4 | 40/368 | 0.94(0.64,1.37) |  | 0.96(0.68,1.44) |  | 0.97(0.66,1.42) |  |
| Dietary nonheme iron |  |  |  |  |  |  |  |
| Q1 | 43/378 | Ref | 0.533 | Ref | 0.590 | Ref | 0.164 |
| Q2 | 42/375 | 0.82(0.52,1.27) |  | 0.78(0.50,1.22) |  | 0.93(0.62,1.41) |  |
| Q3 | 43/376 | 0.94(0.64,1.37) |  | 0.85(0.58,1.25) |  | 0.92(0.57,1.51) |  |
| Q4 | 43/375 | 1.57(1.01,2.43) |  | 1.35(0.86,2.12) |  | 1.86(0.94,3.32) |  |
|  |  |  |  |  |  |  |  |
| **CVD mortality** |  |  |  |  |  |  |  |
| Dietary iron |  |  |  |  |  |  |  |
| Q1 | 17/390 | Ref | 0.561 | Ref | 0.749 | Ref | 0.924 |
| Q2 | 9/371 | 0.61(0.27,1.37) |  | 0.53(0.23,1.22) |  | 0.48(0.20,1.15) |  |
| Q3 | 16/372 | 1.10(0.55,2.21) |  | 1.02(0.50,2.08) |  | 0.92(0.40,2.13) |  |
| Q4 | 14/371 | 1.09(0.53,2.24) |  | 0.97(0.46,2.05) |  | 0.89(0.34,2.35) |  |
| Dietary heme iron |  |  |  |  |  |  |  |
| Q1 | 14/376 | Ref | 0.896 | Ref | 0.809 | Ref | 0.715 |
| Q2 | 15/384 | 0.97(0.49,1.92) |  | 1.03(0.51,2.07) |  | 1.00(0.49,2.04) |  |
| Q3 | 12/376 | 0.83(0.40,1.74) |  | 0.92(0.43,1.97) |  | 0.86(0.39,1.89) |  |
| Q4 | 15/368 | 1.10(0.44,2.73) |  | 0.94(0.37,2.40) |  | 0.91(0.34,2.44) |  |
| Dietary nonheme iron |  |  |  |  |  |  |  |
| Q1 | 14/378 | Ref | 0.631 | Ref | 0.777 | Ref | 0.996 |
| Q2 | 13/375 | 0.93(0.44,1.98) |  | 0.80(0.37,1.73) |  | 0.71(0.31,1.61) |  |
| Q3 | 15/376 | 1.11(0.53,2.32) |  | 1.02(0.48,2.17) |  | 0.93(0.39,2.25) |  |
| Q4 | 14/375 | 1.15(0.54,2.43) |  | 1.04(0.48,2.25) |  | 0.91(0.33,2.47) |  |
|  |  |  |  |  |  |  |  |
| **Diabetes mortality** |  |  |  |  |  |  |  |
| Dietary iron |  |  |  |  |  |  |  |
| Q1 | 9/390 | Ref | 0.005 | Ref | 0.012 | Ref | 0.001 |
| Q2 | 6/371 | 1.11(0.63,3.17) |  | 1.10(0.61,3.20) |  | 1.38(0.75,4.23) |  |
| Q3 | 13/372 | 1.36(0.56,3.31) |  | 1.22(0.48,3.06) |  | 1.85(0.71,4.87) |  |
| Q4 | 15/371 | 1.92(1.73,8.90) |  | 3.06(1.68,6.85) |  | 4.23(3.25,9.20) |  |
| Dietary heme iron |  |  |  |  |  |  |  |
| Q1 | 9/376 | Ref | 0.156 | Ref | 0.186 | Ref | 0.322 |
| Q2 | 7/384 | 0.92(0.37,2.27) |  | 0.98(0.39,2.45) |  | 0.89(0.34,2.30) |  |
| Q3 | 11/376 | 1.01(0.44,2.34) |  | 1.16(0.49,2.73) |  | 1.10(0.46,2.63) |  |
| Q4 | 16/368 | 2.28(0.92,5.67) |  | 2.28(0.89,5.83) |  | 2.03(0.75,5.46) |  |
| Dietary nonheme iron |  |  |  |  |  |  |  |
| Q1 | 9/378 | Ref | 0.111 | Ref | 0.174 | Ref | 0.068 |
| Q2 | 8/375 | 0.84(0.33,2.20) |  | 0.75(0.28,1.98) |  | 0.77(0.28,2.15) |  |
| Q3 | 12/376 | 1.34(0.62,3.10) |  | 1.29(0.53,3.16) |  | 1.58(0.59,4.22) |  |
| Q3 | 14/375 | 1.76(0.75,4.12) |  | 1.56(0.64,3.81) |  | 2.15(0.75,6.17) |  |

Data are HRs and 95%CI; P values were calculated by using multivariate Cox proportional hazards models;

Model 1 adjustment for age, sport, race, education, income;

Model 2 additionally adjustment for BMI, smoke, drink, SBP, DBP, prevalence of hypertension and CVD, serum TC, FPG, fasting insulin, AST, ALT, HDL, LDL, medication for diabetes, hypertension, and dyslipidemia;

Model 3 additionally adjustment for dietary consumption of energy, fiber, fat, protein, carbohydrate, and cholesterol, iron supplement, AHEI.

**Supplement Table 3 HR and 95%CI for the association of dietary total iron, heme iron, and nonheme iron intake with all-cause and disease-specific mortality among diabetes who consumed a daily energy between 450-5000 kcal/d**

| Subgroup | Cases/N | Model 1 | | Model 2 | | Model 3 | |
| --- | --- | --- | --- | --- | --- | --- | --- |
|  |  | OR(95%CI) | P | OR(95%CI) | P | OR(95%CI) | P |
| **Men** |  |  |  |  |  |  |  |
| **All-cause** |  |  |  |  |  |  |  |
| Dietary iron |  |  |  |  |  |  |  |
| Q1 | 159/613 | Ref | 0.016 | Ref | 0.020 | Ref | 0.240 |
| Q2 | 123/595 | 0.80(0.63,1.01) |  | 0.80(0.63,1.02) |  | 0.86(0.66,1.10) |  |
| Q3 | 127/595 | 0.88(0.69,1.11) |  | 0.91(0.72,1.16) |  | 0.95(0.72,1.27) |  |
| Q4 | 109/595 | 0.70(0.55,0.90) |  | 0.70(0.54,0.90) |  | 0.78(0.56,1.08) |  |
| Dietary heme iron |  |  |  |  |  |  |  |
| Q1 | 116/603 | Ref | 0.016 | Ref | 0.038 | Ref | 0.016 |
| Q2 | 130/607 | 1.12(0.82,1.53) |  | 1.15(0.87,1.44) |  | 1.16(0.87,1.46) |  |
| Q3 | 137/599 | 1.18(0.87,1.61) |  | 1.20(0.90,1.50) |  | 1.22(0.91,1.52) |  |
| Q4 | 135/588 | 1.37(1.02,1.83) |  | 1.28(0.96,1.72) |  | 1.35(1.01,1.82) |  |
| Dietary nonheme iron |  |  |  |  |  |  |  |
| Q1 | 154/608 | Ref | 0.006 | Ref | 0.013 | Ref | 0.137 |
| Q2 | 138/605 | 0.86(0.68,1.08) |  | 0.88(0.70,1.11) |  | 0.92(0.72,1.18) |  |
| Q3 | 120/599 | 0.83(0.65,1.06) |  | 0.87(0.68,1.11) |  | 0.89(0.67,1.18) |  |
| Q4 | 106/586 | 0.70(0.55,0.90) |  | 0.72(0.56,0.92) |  | 0.78(0.57,1.07) |  |
|  |  |  |  |  |  |  |  |
| **CVD mortality** |  |  |  |  |  |  |  |
| Dietary iron |  |  |  |  |  |  |  |
| Q1 | 62/613 | Ref | 0.034 | Ref | 0.065 | Ref | 0.259 |
| Q2 | 44/595 | 0.73(0.49,1.07) |  | 0.72(0.48,1.06) |  | 0.76(0.50,1.16) |  |
| Q3 | 41/595 | 0.77(0.52,1.15) |  | 0.85(0.56,1.28) |  | 0.90(0.55,1.46) |  |
| Q4 | 35/595 | 0.61(0.40,0.94) |  | 0.63(0.41,0.96) |  | 0.67(0.39,1.18) |  |
| Dietary heme iron |  |  |  |  |  |  |  |
| Q1 | 35/603 | Ref | 0.037 | Ref | 0.060 | Ref | 0.044 |
| Q2 | 48/607 | 1.35(0.76,2.37) |  | 1.28(0.72,2.28) |  | 1.23(0.69,2.20) |  |
| Q3 | 51/599 | 1.56(0.89,2.72) |  | 1.43(0.92,2.51) |  | 1.38(0.96,2.44) |  |
| Q4 | 48/588 | 1.70(1.01,2.90) |  | 1.58(1.01,2.70) |  | 1.59(1.02,2.73) |  |
| Dietary nonheme iron |  |  |  |  |  |  |  |
| Q1 | 59/608 | Ref | 0.022 | Ref | 0.057 | Ref | 0.215 |
| Q2 | 46/605 | 0.75(0.51,1.10) |  | 0.77(0.52,1.14) |  | 0.82(0.54,1.24) |  |
| Q3 | 46/599 | 0.85(0.57,1.25) |  | 0.94(0.63,1.39) |  | 0.98(0.62,1.55) |  |
| Q4 | 31/586 | 0.56(0.36,0.87) |  | 0.59(0.38,0.93) |  | 0.64(0.37,1.11) |  |
|  |  |  |  |  |  |  |  |
| **Diabetes mortality** |  |  |  |  |  |  |  |
| Dietary iron |  |  |  |  |  |  |  |
| Q1 | 56/613 | Ref | 0.013 | Ref | 0.016 | Ref | 0.400 |
| Q2 | 34/595 | 0.67(0.44,1.03) |  | 0.71(0.46,1.08) |  | 0.73(0.46,1.16) |  |
| Q3 | 39/595 | 0.77(0.51,1.17) |  | 0.80(0.52,1.23) |  | 0.85(0.50,1.43) |  |
| Q4 | 26/595 | 0.51(0.32,0.83) |  | 0.52(0.32,0.84) |  | 0.57(0.31,1.08) |  |
| Dietary heme iron |  |  |  |  |  |  |  |
| Q1 | 37/603 | Ref | 0.956 | Ref | 0.918 | Ref | 0.841 |
| Q2 | 42/607 | 1.29(0.74,2.24) |  | 1.22(0.70,2.15) |  | 1.26(0.71,2.22) |  |
| Q3 | 39/599 | 1.12(0.62,1.92) |  | 1.07(0.60,1.89) |  | 1.15(0.62,1.98) |  |
| Q4 | 37/588 | 1.15(0.67,1.97) |  | 1.09(0.63,1.87) |  | 1.17(0.68,2.03) |  |
| Dietary nonheme iron |  |  |  |  |  |  |  |
| Q1 | 51/608 | Ref | 0.053 | Ref | 0.075 | Ref | 0.493 |
| Q2 | 38/605 | 0.73(0.48,1.12) |  | 0.76(0.50,1.17) |  | 0.80(0.51,1.27) |  |
| Q3 | 39/599 | 0.83(0.55,1.27) |  | 0.89(0.58,1.36) |  | 0.98(0.59,1.62) |  |
| Q4 | 27/586 | 0.59(0.37,0.94) |  | 0.60(0.37,0.97) |  | 0.74(0.41,1.35) |  |
|  |  |  |  |  |  |  |  |
| **Women** |  |  |  |  |  |  |  |
| **All-cause** |  |  |  |  |  |  |  |
| Dietary iron |  |  |  |  |  |  |  |
| Q1 | 96/520 | Ref | 0.807 | Ref | 0.545 | Ref | 0.108 |
| Q2 | 80/488 | 0.94(0.72,1.23) |  | 0.86(0.72,1.24) |  | 0.97(0.71,1.33) |  |
| Q3 | 69/488 | 0.83(0.68,1.26) |  | 0.85(0.72,1.34) |  | 1.01(0.74,1.45) |  |
| Q4 | 86/488 | 1.07(0.98,1.92) |  | 1.12(0.91,1.61) |  | 1.35(0.95,1.92) |  |
| Dietary heme iron |  |  |  |  |  |  |  |
| Q1 | 89/492 | Ref | 0.361 | Ref | 0.399 | Ref | 0.833 |
| Q2 | 92/520 | 0.90(0.69,1.19) |  | 0.92(0.70,1.22) |  | 0.95(0.72,1.26) |  |
| Q3 | 68/485 | 0.67(0.49,0.92) |  | 0.73(0.53,1.01) |  | 0.77(0.56,1.07) |  |
| Q4 | 82/487 | 0.95(0.70,1.28) |  | 0.96(0.70,1.43) |  | 1.05(0.77,1.45) |  |
| Dietary nonheme iron |  |  |  |  |  |  |  |
| Q1 | 87/499 | Ref | 0.801 | Ref | 0.501 | Ref | 0.095 |
| Q2 | 81/495 | 0.88(0.65,1.19) |  | 0.84(0.62,1.14) |  | 0.94(0.68,1.29) |  |
| Q3 | 78/495 | 0.88(0.64,1.19) |  | 0.88(0.65,1.20) |  | 1.06(0.75,1.50) |  |
| Q4 | 85/495 | 1.05(0.77,1.41) |  | 1.11(0.82,1.50) |  | 1.34(0.94,1.92) |  |
|  |  |  |  |  |  |  |  |
| **CVD mortality** |  |  |  |  |  |  |  |
| Dietary iron |  |  |  |  |  |  |  |
| Q1 | 38/520 | Ref | 0.971 | Ref | 0.815 | Ref | 0.569 |
| Q2 | 24/488 | 0.71(0.42,1.18) |  | 0.69(0.41,1.16) |  | 0.79(0.46,1.36) |  |
| Q3 | 27/488 | 0.81(0.49,1.34) |  | 0.88(0.53,1.46) |  | 1.04(0.60,1.82) |  |
| Q4 | 28/488 | 0.89(0.55,1.47) |  | 1.04(0.63,1.71) |  | 1.14(0.63,2.06) |  |
| Dietary heme iron |  |  |  |  |  |  |  |
| Q1 | 32/492 | Ref | 0.339 | Ref | 0.388 | Ref | 0.589 |
| Q2 | 36/520 | 0.97(0.56,1.40) |  | 1.01(0.58,1.45) |  | 1.02(0.68,1.49) |  |
| Q3 | 22/485 | 0.69(0.41,1.16) |  | 0.67(0.45,1.30) |  | 0.69(0.47,1.36) |  |
| Q4 | 27/487 | 0.89(0.48,1.65) |  | 0.88(0.52,1.58) |  | 0.94(0.49,1.81) |  |
| Dietary nonheme iron |  |  |  |  |  |  |  |
| Q1 | 34/499 | Ref | 0.940 | Ref | 0.949 | Ref | 0.849 |
| Q2 | 28/495 | 0.78(0.47,1.29) |  | 0.72(0.49,1.20) |  | 0.78(0.43,1.33) |  |
| Q3 | 29/495 | 0.82(0.50,1.36) |  | 0.87(0.52,1.44) |  | 1.02(0.51,1.81) |  |
| Q4 | 26/495 | 0.81(0.49,1.36) |  | 0.94(0.56,1.59) |  | 0.99(0.53,1.85) |  |
|  |  |  |  |  |  |  |  |
| **Diabetes mortality** |  |  |  |  |  |  |  |
| Dietary iron |  |  |  |  |  |  |  |
| Q1 | 23/520 | Ref | 0.053 | Ref | 0.026 | Ref | 0.004 |
| Q2 | 20/488 | 0.91(0.50,1.67) |  | 0.83(0.45,1.52) |  | 0.92(0.49,1.73) |  |
| Q3 | 22/488 | 1.05(0.58,1.90) |  | 1.14(0.63,2.07) |  | 1.44(0.76,2.71) |  |
| Q4 | 32/488 | 1.66(0.97,2.86) |  | 1.75(1.01,3.01) |  | 2.31(1.23,4.32) |  |
| Dietary heme iron |  |  |  |  |  |  |  |
| Q1 | 17/492 | Ref | 0.080 | Ref | 0.077 | Ref | 0.082 |
| Q2 | 27/520 | 1.36(0.74,2.50) |  | 1.41(0.76,2.62) |  | 1.45(0.78,2.70) |  |
| Q3 | 22/485 | 1.13(0.60,2.14) |  | 1.24(0.66,2.36) |  | 1.27(0.67,2.43) |  |
| Q4 | 31/487 | 1.83(1.01,3.31) |  | 1.84(1.01,3.36) |  | 1.87(1.00,3.49) |  |
| Dietary nonheme iron |  |  |  |  |  |  |  |
| Q1 | 21/499 | Ref | 0.128 | Ref | 0.046 | Ref | 0.007 |
| Q2 | 21/495 | 0.92(0.50,1.69) |  | 0.86(0.47,1.60) |  | 0.98(0.52,1.85) |  |
| Q3 | 26/495 | 1.19(0.66,2.12) |  | 1.28(0.71,2.30) |  | 1.63(0.87,3.08) |  |
| Q4 | 29/495 | 1.45(0.83,2.56) |  | 1.62(0.91,2.88) |  | 2.21(1.14,4.27) |  |

Data are HRs and 95%CI; P values were calculated by using multivariate Cox proportional hazards models;

Model 1 adjustment for age, sport, race, education, income;

Model 2 additionally adjustment for BMI, smoke, drink, SBP, DBP, prevalence of hypertension and CVD, serum TC, FPG, fasting insulin, AST, ALT, HDL, LDL, medication for diabetes, hypertension, and dyslipidemia;

Model 3 additionally adjustment for dietary consumption of energy, fiber, fat, protein, carbohydrate, and cholesterol, iron supplement, AHEI.

**Supplement Table 4 HR and 95%CI for the association of dietary total iron, heme iron, and nonheme iron intake with all-cause and disease-specific mortality among diabetes without iron supplement**

| Subgroup | Cases/N | Model 1 | | Model 2 | | Model 3 | |
| --- | --- | --- | --- | --- | --- | --- | --- |
|  |  | HR(95%CI) | P | HR(95%CI) | P | HR(95%CI) | P |
| **Men** |  |  |  |  |  |  |  |
| **All-cause** |  |  |  |  |  |  |  |
| Dietary iron |  |  |  |  |  |  |  |
| Q1 | 148/565 | Ref | 0.004 | Ref | 0.005 | Ref | 0.134 |
| Q2 | 107/535 | 0.73(0.56,0.93) |  | 0.73(0.57,0.95) |  | 0.78(0.59,1.02) |  |
| Q3 | 111/519 | 0.85(0.66,1.09) |  | 0.88(0.68,1.13) |  | 0.92(0.67,1.25) |  |
| Q4 | 86/510 | 0.63(0.48,0.82) |  | 0.63(0.48,0.83) |  | 0.70(0.49,1.00) |  |
| Dietary heme iron |  |  |  |  |  |  |  |
| Q1 | 99/534 | Ref | 0.016 | Ref | 0.038 | Ref | 0.023 |
| Q2 | 117/538 | 1.33(1.02,1.74) |  | 1.35(1.02,1.77) |  | 1.32(1.00,1.74) |  |
| Q3 | 118/533 | 1.27(0.97,1.66) |  | 1.17(0.89,1.53) |  | 1.23(0.93,1.63) |  |
| Q4 | 118/524 | 1.44(1.10,1.88) |  | 1.42(1.08,1.86) |  | 1.43(1.09,1.89) |  |
| Dietary nonheme iron |  |  |  |  |  |  |  |
| Q1 | 142/558 | Ref | 0.002 | Ref | 0.004 | Ref | 0.073 |
| Q2 | 119/535 | 0.81(0.64,1.04) |  | 0.83(0.65,1.07) |  | 0.85(0.65,1.11) |  |
| Q3 | 103/524 | 0.78(0.61,1.01) |  | 0.82(0.63,1.06) |  | 0.84(0.62,1.13) |  |
| Q4 | 88/512 | 0.65(0.49,0.85) |  | 0.66(0.50,0.86) |  | 0.72(0.51,1.01) |  |
|  |  |  |  |  |  |  |  |
| **CVD mortality** |  |  |  |  |  |  |  |
| Dietary iron |  |  |  |  |  |  |  |
| Q1 | 58/565 | Ref | 0.029 | Ref | 0.053 | Ref | 0.160 |
| Q2 | 37/535 | 0.66(0.43,0.99) |  | 0.64(0.42,0.97) |  | 0.63(0.40,1.00) |  |
| Q3 | 38/519 | 0.78(0.51,1.17) |  | 0.85(0.55,1.30) |  | 0.84(0.50,1.42) |  |
| Q4 | 29/510 | 0.57(0.36,0.90) |  | 0.58(0.37,0.92) |  | 0.57(0.31,1.05) |  |
| Dietary heme iron |  |  |  |  |  |  |  |
| Q1 | 33/534 | Ref | 0.125 | Ref | 0.238 | Ref | 0.173 |
| Q2 | 44/538 | 1.44(0.91,2.26) |  | 1.37(0.86,2.17) |  | 1.36(0.85,2.17) |  |
| Q3 | 44/533 | 1.44(0.91,2.26) |  | 1.27(0.81,2.01) |  | 1.38(0.86,2.21) |  |
| Q4 | 41/524 | 1.47(0.93,2.32) |  | 1.39(0.87,2.20) |  | 1.42(0.88,2.26) |  |
| Dietary nonheme iron |  |  |  |  |  |  |  |
| Q1 | 55/558 | Ref | 0.020 | Ref | 0.048 | Ref | 0.126 |
| Q2 | 40/535 | 0.71(0.47,1.07) |  | 0.72(0.48,1.09) |  | 0.71(0.45,1.11) |  |
| Q3 | 40/524 | 0.81(0.54,1.22) |  | 0.89(0.59,1.36) |  | 0.89(0.54,1.47) |  |
| Q4 | 27/512 | 0.54(0.34,0.86) |  | 0.57(0.36,0.91) |  | 0.55(0.30,1.01) |  |
|  |  |  |  |  |  |  |  |
| **Diabetes mortality** |  |  |  |  |  |  |  |
| Dietary iron |  |  |  |  |  |  |  |
| Q1 | 50/565 | Ref | 0.016 | Ref | 0.018 | Ref | 0.133 |
| Q2 | 30/535 | 0.62(0.39,0.98) |  | 0.63(0.40,1.00) |  | 0.63(0.38,1.04) |  |
| Q3 | 33/519 | 0.77(0.49,1.20) |  | 0.81(0.51,1.29) |  | 0.85(0.48,1.51) |  |
| Q4 | 21/510 | 0.49(0.29,0.82) |  | 0.48(0.28,0.82) |  | 0.51(0.26,1.02) |  |
| Dietary heme iron |  |  |  |  |  |  |  |
| Q1 | 30/534 | Ref | 0.408 | Ref | 0.635 | Ref | 0.461 |
| Q2 | 36/538 | 1.29(0.79,2.10) |  | 1.26(0.77,2.07) |  | 1.28(0.78,2.10) |  |
| Q3 | 34/533 | 1.20(0.79,2.10) |  | 1.09(0.66,1.78) |  | 1.23(0.74,2.06) |  |
| Q4 | 34/524 | 1.28(0.78,2.09) |  | 1.20(0.73,1.97) |  | 1.24(0.75,2.06) |  |
| Dietary nonheme iron |  |  |  |  |  |  |  |
| Q1 | 46/558 | Ref | 0.061 | Ref | 0.079 | Ref | 0.451 |
| Q2 | 31/535 | 0.68(0.43,1.07) |  | 0.69(0.44,1.10) |  | 0.70(0.42,1.16) |  |
| Q3 | 34/524 | 0.82(0.53,1.28) |  | 0.88(0.56,1.38) |  | 0.96(0.55,1.66) |  |
| Q4 | 23/512 | 0.57(0.34,0.95) |  | 0.58(0.34,0.96) |  | 0.68(0.35,1.32) |  |
|  |  |  |  |  |  |  |  |
| **Women** |  |  |  |  |  |  |  |
| **All-cause** |  |  |  |  |  |  |  |
| Dietary iron |  |  |  |  |  |  |  |
| Q1 | 85/445 | Ref | 0.786 | Ref | 0.578 | Ref | 0.089 |
| Q2 | 66/413 | 0.81(0.59,1.12) |  | 0.76(0.55,1.06) |  | 0.88(0.63,1.24) |  |
| Q3 | 64/415 | 0.87(0.62,1.20) |  | 0.90(0.65,1.25) |  | 1.12(0.78,1.61) |  |
| Q4 | 70/414 | 1.05(0.76,1.44) |  | 1.08(0.78,1.49) |  | 1.35(0.92,1.99) |  |
| Dietary heme iron |  |  |  |  |  |  |  |
| Q1 | 80/419 | Ref | 0.070 | Ref | 0.110 | Ref | 0.339 |
| Q2 | 75/433 | 0.84(0.61,1.16) |  | 0.84(0.61,1.17) |  | 0.88(0.63,1.22) |  |
| Q3 | 65/418 | 0.72(0.52,1.01) |  | 0.77(0.55,1.07) |  | 0.81(0.58,1.14) |  |
| Q4 | 65/417 | 0.77(0.55,1.07) |  | 0.78(0.56,1.09) |  | 0.87(0.61,1.23) |  |
| Dietary nonheme iron |  |  |  |  |  |  |  |
| Q1 | 74/423 | Ref | 0.580 | Ref | 0.367 | Ref | 0.038 |
| Q2 | 72/421 | 0.90(0.65,1.24) |  | 0.84(0.61,1.17) |  | 0.97(0.69,1.36) |  |
| Q3 | 66/422 | 0.88(0.63,1.22) |  | 0.89(0.63,1.25) |  | 1.13(0.78,1.63) |  |
| Q4 | 73/421 | 1.12(0.80,1.55) |  | 1.17(0.84,1.63) |  | 1.49(1.01,2.20) |  |
|  |  |  |  |  |  |  |  |
| **CVD mortality** |  |  |  |  |  |  |  |
| Dietary iron |  |  |  |  |  |  |  |
| Q1 | 31/445 | Ref | 0.663 | Ref | 0.322 | Ref | 0.213 |
| Q2 | 19/413 | 0.65(0.37,1.15) |  | 0.65(0.36,1.16) |  | 0.74(0.40,1.36) |  |
| Q3 | 26/415 | 0.98(0.58,1.67) |  | 1.05(0.61,1.80) |  | 1.35(0.74,2.46) |  |
| Q4 | 24/414 | 1.04(0.60,1.79) |  | 1.22(0.700,2.12) |  | 1.35(0.70,2.62) |  |
| Dietary heme iron |  |  |  |  |  |  |  |
| Q1 | 28/419 | Ref | 0.244 | Ref | 0.299 | Ref | 0.427 |
| Q2 | 28/433 | 0.89(0.52,1.51) |  | 0.94(0.55,1.61) |  | 0.98(0.56,1.70) |  |
| Q3 | 21/418 | 0.66(0.38,1.17) |  | 0.73(0.41,1.29) |  | 0.73(0.41,1.31) |  |
| Q4 | 23/417 | 0.78(0.45,1.36) |  | 0.80(0.45,1.40) |  | 0.87(0.48,1.56) |  |
| Dietary nonheme iron |  |  |  |  |  |  |  |
| Q1 | 26/423 | Ref | 0.759 | Ref | 0.367 | Ref | 0.272 |
| Q2 | 25/421 | 0.88(0.51,1.53) |  | 0.81(0.47,1.42) |  | 0.91(0.51,1.64) |  |
| Q3 | 25/422 | 0.96(0.55,1.67) |  | 1.00(0.57,1.76) |  | 1.26(0.67,2.37) |  |
| Q4 | 24/421 | 1.07(0.61,1.88) |  | 1.26(0.71,2.23) |  | 1.38(0.70,2.72) |  |
|  |  |  |  |  |  |  |  |
| **Diabetes mortality** |  |  |  |  |  |  |  |
| Dietary iron |  |  |  |  |  |  |  |
| Q1 | 19/445 | Ref | 0.065 | Ref | 0.031 | Ref | 0.007 |
| Q2 | 16/413 | 0.86(0.44,1.67) |  | 0.78(0.40,1.54) |  | 0.90(0.45,1.83) |  |
| Q3 | 21/415 | 1.23(0.66,2.31) |  | 1.42(0.75,2.69) |  | 1.85(0.93,3.68) |  |
| Q4 | 25/414 | 1.64(0.89,3.01) |  | 1.72(0.93,3.19) |  | 2.27(1.11,4.66) |  |
| Dietary heme iron |  |  |  |  |  |  |  |
| Q1 | 14/419 | Ref | 0.121 | Ref | 0.108 | Ref | 0.101 |
| Q2 | 19/433 | 1.19(0.59,2.38) |  | 1.17(0.58,2.38) |  | 1.21(0.60,2.47) |  |
| Q3 | 23/418 | 1.44(0.74,2.81) |  | 1.55(0.79,3.05) |  | 1.63(0.82,3.23) |  |
| Q4 | 25/417 | 1.61(0.83,3.11) |  | 1.61(0.83,3.13) |  | 1.67(0.84,3.32) |  |
| Dietary nonheme iron |  |  |  |  |  |  |  |
| Q1 | 17/423 | Ref | 0.072 | Ref | 0.026 | Ref | 0.004 |
| Q2 | 17/421 | 0.91(0.46,1.79) |  | 0.84(0.42,1.66) |  | 0.98(0.48,1.99) |  |
| Q3 | 22/422 | 1.25(0.66,2.37) |  | 1.36(0.71,2.60) |  | 1.83(0.90,3.69) |  |
| Q4 | 25/421 | 1.64(0.88,3.06) |  | 1.82(0.97,3.43) |  | 2.60(1.26,5.39) |  |

Data are HRs and 95%CI; P values were calculated by using multivariate Cox proportional hazards models;

Model 1 adjustment for age, sport, race, education, income;

Model 2 additionally adjustment for BMI, smoke, drink, SBP, DBP, prevalence of hypertension and CVD, serum TC, FPG, fasting insulin, AST, ALT, HDL, LDL, medication for diabetes, hypertension, and dyslipidemia;

Model 3 additionally adjustment for dietary consumption of energy, fiber, fat, protein, carbohydrate, and cholesterol, AHEI.
